# Dyslipidemia induced large-scale network connectivity abnormality facilitates cognitive decline in the Alzheimer’s disease

**DOI:** 10.1186/s12967-022-03786-w

**Published:** 2022-12-06

**Authors:** Qing Wang, Feifei Zang, Cancan He, Zhijun Zhang, Chunming Xie, Alzheimer’s Disease Neuroimaging Initiative

**Affiliations:** 1 Department of Neurology, Affiliated ZhongDa Hospital, School of Medicine, Southeast University, Nanjing, 210009 Jiangsu China; 2 Institute of Neuropsychiatry, Affiliated ZhongDa Hospital, Southeast University, Nanjing, 210009 Jiangsu China; 3 The Key Laboratory of Developmental Genes and Human Disease, Southeast University, Nanjing, 210009 Jiangsu China

**Keywords:** Alzheimer’s disease, Lipid, Large-scale network, Canonical correlation analysis

## Abstract

**Background:**

Although lipid metabolite dysfunction contributes substantially to clinical signs and pathophysiology of Alzheimer’s disease (AD), how dyslipidemia promoting neuropathological processes and brain functional impairment subsequently facilitates the progression of AD remains unclear.

**Methods:**

We combined large-scale brain resting-state networks (RSNs) approaches with canonical correlation analysis to explore the accumulating effects of lipid gene- and protein-centric levels on cerebrospinal fluid (CSF) biomarkers, dynamic trajectory of large-scale RSNs, and cognitive performance across entire AD spectrum. Support vector machine model was used to distinguish AD spectrum and pathway analysis was used to test the influences among these variables.

**Results:**

We found that the effects of accumulation of lipid-pathway genetic variants and lipoproteins were significantly correlated with CSF biomarkers levels and cognitive performance across the AD spectrum. Dynamic trajectory of large-scale RSNs represented a rebounding mode, which is characterized by a weakened network cohesive connector role and enhanced network incohesive provincial role following disease progression. Importantly, the fluctuating large-scale RSNs connectivity was significantly correlated with the summative effects of lipid-pathway genetic variants and lipoproteins, CSF biomarkers, and cognitive performance. Moreover, SVM model revealed that the lipid-associated twenty-two brain network connections represented higher capacity to classify AD spectrum. Pathway analysis further identified dyslipidemia directly influenced brain network reorganization or indirectly affected the CSF biomarkers and subsequently caused cognitive decline.

**Conclusions:**

Dyslipidemia exacerbated cognitive decline and increased the risk of AD via mediating large-scale brain networks integrity and promoting neuropathological processes.

These findings reveal a role for lipid metabolism in AD pathogenesis and suggest lipid management as a potential therapeutic target for AD.

**Supplementary Information:**

The online version contains supplementary material available at 10.1186/s12967-022-03786-w.

## Background

Lipids are important components of the brain that play a critical role in the membrane formation of neuronal cells, and participate in essential physiological functions such as cellular transport, energy storage, in addition to acting to modulate transmembrane proteins and signaling molecules, promoting effective signal transduction, and regulating gene expression [1–3]. In recent years, growing evidence from both animal models and humans’ studies has identified that abnormal lipid metabolites were associated with the molecular mechanisms underlying Alzheimer’s disease (AD) pathophysiology beyond amyloid plaques and neurofibrillary tangles [4–9]. In fact, altered plasma lipid profiles have appeared to exacerbate cognitive decline, subsequently increasing the risk of the incidence of AD in nondemented elderly adults [7, 10–14]. Specifically, recent biological and neuroimaging data have indicated that the dysfunctional composition of lipid rafts, primarily located in membrane microdomains and serving as an important platform for signal processing, may contribute to AD pathophysiology [2, 11]. Cholesterol, as a major component of lipid rafts, is thought to be involved in amyloid precursor protein (APP) processing and β-amyloid (Aβ) overproduction characterized as a key feature of AD pathophysiology [12], while gemfibrozil, a fibric acid agent commonly used to treat hyperlipidemias in clinic, significantly reduces amyloid pathology and reverses memory deficits in APP-PSEN1ΔE9 mice [15], a murine model that mimics AD-like pathology and cognitive decline. As changes of lipoprotein in the blood can be detected prior to cognitive decline, it is of considerable interest to know whether lipid pathway-based metabolites substantially contribute to AD pathophysiology [16]. However, to date, it remains unclear how lipid metabolites, cerebral spinal fluid (CSF) biomarkers, and brain function are linked or interacted with the progression of cognitive decline in preclinical or clinical AD patients.

Brain network integrity plays an instrumental role in the regulation of high-order cognitive function. Resting-state networks (RSNs), which measure temporal correlation depend on intrinsic blood oxygenation level dependent (BOLD) signals within large-scale systems and provide a powerful tool to investigate network integrity between structurally segregated and functionally specialized brain regions at the system level [17]. Importantly, the spatial–temporal evolution of RSNs has been found to be tightly associated with neural correlates of cognitive impairment observed in preclinical and clinical AD patients [18–21], including default mode network (DMN), executive control network (ECN), salience network (SAN), attention network (AN), and visuospatial network (VIS), suggesting that changes in distributed networks at a large-scale system level could predict clinical progression and neurodegeneration [18, 22, 23]. Recently, particular attention to network integrity has shifted towards investigation of molecular pathological changes invoked in intrinsic large-scale network dynamics supporting diverse cognitive function [24]. Specifically, increasing evidence has demonstrated that neural correlates of the disrupted connectivity of RSNs in cognitively healthy individuals with brain amyloidosis or AD-related genetic risk factors were similar to abnormalities observed in symptomatic AD [25–27]. As such, it may be possible that RSNs could be used as an intermediate phenotype linking downstream cognitive decline and upstream molecular cascading events underlying AD pathophysiology. Dysregulation of lipids is substantially associated with the disrupted architecture of RSNs and is directly involved in the molecular and cellular changes underlying AD pathophysiology [28, 29]. For example, high serum cholesterol has been associated with decreased cortical and hippocampal volumes in cholesterol-fed rabbits [30] and disrupt functional connectivity of the SAN in the non-demented elderly [28]. Increased low-density lipoprotein cholesterol (LDL-C) levels causes a detrimental effect to posterior cingulate gray matter volumes and verbal memory [31], while elevated high-density lipoprotein cholesterol (HDL-C) provides protection against hippocampal atrophy and AD [32, 33]. From our previous work, we previously reported that the effects of the accumulation of genetic variants of cholesterol-pathway molecules produces widespread effects on cortico-subcortical-cerebellar spontaneous brain activity in amnestic mild cognitive impairment (aMCI) patients [34]. These findings suggest that several, distinct lipidomic signatures influence brain network integrity and subsequently contribute to AD. However, it remains unclear how altered lipid metabolites impinge on the dynamic spatiotemporal patterns of RSNs as AD progresses. Indeed, in the context of lipid-centric gene and protein changes, evaluation of the potential effects of lipid abnormalities that affect dynamic brain network trajectory and CSF biomarkers, subsequently leading to cognitive decline, are beneficial in order to capture a more holistic picture of the processes of AD.

In the present study, a new approach was used that combining large-scale brain networks with canonical correlation analysis (CCA) to explore the effects of lipid metabolic disturbance on the dynamic trajectory of ten RSNs changes and molecular biomarkers that promote cognitive decline following AD progression. Firstly, the relationship between lipid-centric gene variants and proteins, CSF biomarkers, and cognitive performance across the AD spectrum (ADS) was examined. Secondly, the dynamic trajectory of large-scale network changes was identified both within- and between ten predefined RSNs from cognitive normal (CN) healthy to mild AD stage individuals. Thirdly, the potential associations between lipid-related gene variants and proteins, and the dynamic trajectory of large-scale network connectivity, CSF biomarkers, and cognitive performance were explored using CCA. Fourth, a support vector machine (SVM) model of machine learning was used to distinguish ADS patients from CN subjects. Finally, path analysis with structural equation modeling (SEM) was used to test the effects of lipoproteins on large-scale RSNs, CSF biomarkers, and cognitive performance. Taken together, these findings provided an integrated view of lipid metabolite abnormalities exacerbated cognitive decline and increased the risk of AD occurrence via mediating large-scale brain network integrity and promoting neuropathological processes.

## Methods

### Participants

All data at baseline were extracted from the Alzheimer's disease Neuroimaging Initiative (ADNI) database (http://adni.loni.usc.edu) prior to January 20th, 2020. Data for lipid gene and protein expression, CSF biomarkers and that of imaging quality control of a total of 124 subjects incorporating 51 cognitive normal (CN), 26 early amnestic mild cognitive impairment (EMCI), 26 late mild cognitive impairment (LMCI) and 21 mild Alzheimer’s disease (AD) subjects were included in the present study (Table 1). Detailed inclusion and exclusion criteria were provided in Additional file 1.

**Table 1:** Demographic data, lipid pathway-based genotypes, cerebrospinal fluid biomarkers, and global cognitive performance across the AD spectrum

Items	CN	EMCI	LMCI	AD	P values
	(n = 51)	(n = 26)	(n = 26)	(n = 21)	
Age (years)	74.08 ± 5.79	70.04 ± 6.87	70.81 ± 7.14	71.81 ± 7.77	0.051
Gender (F/M)	30/21	14/12	11/15	9/12	0.447*
Education (years)	16.31 ± 2.59	15.27 ± 2.51	16.31 ± 2.51	15.14 ± 2.76	0.157
Multiple protective genes					
CLU T status (TC + TT/CC)	38/13	17/9	18/8	13/8	0.710*
LDLR A status (AG + AA/GG)	38/13	16/10	15/11	11/10	0.240*
LRP1 T status (TC + TT/CC)	15/36	8/18	5/21	10/11	0.214*
PICALM A status (AG + AA/GG)	27/24	15/11	16/10	11/10	0.884*
Multiple risk genes					
APOE ε4 status (+ / −)	15/36	14/12	12/14	15/6	0.008*
SORL1 G status (TG + GG/TT)	19/32	12/14	12/14	6/15	0.548*
CETP A status (AG + AA/GG)	46/5	24/2	23/3	19/2	0.974*
ABCA1 G status (AG + GG/AA)	45/6	25/1	21/5	19/2	0.369*
BIN1 C status (TC + CC/TT)	27/24	17/9	15/11	11/10	0.739*
Cerebrospinal fluid biomarkers					
Aβ (pg/ml)	192.79 ± 50.17^bc^	183.61 ± 50.66^d^	168.77 ± 50.80	140.40 ± 43.59	0.001
Tau (pg/ml)	68.53 ± 34.14^c^	79.32 ± 51.89^d^	86.01 ± 52.19^e^	129.29 ± 61.42	< 0.001
pTau (pg/ml)	34.18 ± 16.58^bc^	39.60 ± 24.73^d^	48.42 ± 33.50	55.23 ± 26.13	0.005
Global cognitive performance					
MMSE	28.84 ± 1.16^abc^	27.92 ± 2.13^d^	27.73 ± 1.54^e^	22.67 ± 2.50	< 0.001
ADAS-Cog	10.76 ± 6.53^abc^	14.19 ± 6.58^d^	16.96 ± 5.32^e^	35.81 ± 8.99	< 0.001

*, p values were obtained using a Chi-square test; other p values were obtained from a one-way ANOVA. Unless indicated, data are presented as means ± standard deviation. Post hoc analyses were used with least significance difference correction (p < 0.05): a: statistical difference detected between CN group and EMCI group; b: statistical difference was detected between CN group and LMCI group; c: statistical difference was detected between CN group and AD group; d: statistical difference was detected between EMCI group and AD group; e: statistical difference was detected between LMCI group and AD group. *CN* cognitively normal, *EMCI* early mild cognitive impairment, *LMCI* late mild cognitive impairment, *AD* Alzheimer’s disease, *M/F* male/female, *CLU* clusterin, *LDLR* low density lipoprotein receptor, *LRP1* low density lipoprotein receptor-related protein 1, *PICALM* phosphatidylinositol-binding clathrin assembly protein, *APOE* apolipoprotein E, *SORL1* sortilin-related receptor 1, *CETP* cholesterol ester transfer protein, *ABCA1* ATP-binding cassette transporter A1; *BIN1* bridging integrator 1, *Aβ* amyloid-1 to 42 peptide, *Tau* total tau, *pTau* tau phosphorylated at the threonine 181 position, *MMSE* mini-mental state examination, *ADAS-Cog* Alzheimer’s Disease Assessment Scale-Cognitive Subscale

Demographic data such as age, gender and years of education were enrolled in this study. Based on the cholesterol metabolism pathway, nine candidate genes were selected: CLU, LDLR, LRP1, PICALM, SORL1, CETP, ABCA1, BIN1 and APOE (Tables 1 and 2). Hardy–Weinberg equilibrium test for each allele was calculated with chi-square test. In addition, thirty-eight lipid metabolic biomarkers were obtained. The detailed acquisition and selection procedures of plasma lipids were available in the Additional file 1. Further, CSF biomarkers including Amyloid-β 1 to 42 peptide (Aβ), total tau (Tau) and tau phosphorylated at the threonine 181 (pTau) were collected. The MMSE and Alzheimer’s Disease Assessment Scale-Cognitive Subscale (ADAS-Cog) were used to measuring global cognitive function.

### Calculation of polygenic scores

Genes were divided into two categories: protective or hazardous, depending on the value of odds ratio (OR) for each gene. For OR values > 1, the locus was defined as hazardous, while for OR < 1 there were defined as protective variants. Polygenic scores were defined as a sum of ORs of multiple loci. The concept of relative risk score (RRS) utilized in the present study was defined as genetic risk score (GRS) minus genetic protective score (GPS). Due to the strong risk effect of the APOE genotype, GRS was calculated with the APOEε4 (GRS) and without the APOEε4 genotype (GRS_n), respectively. Consequently, RRS was also separated into RRS with APOEε4 (RRS) and RRS without APOEε4 (RRS_n). Gene information was detailed in Additional file 1: Table S1.

### Functional network construction

Resting-state functional MRI image acquisition and preprocessing procedures were described in Additional file 1. The atlas of Power et al. [35] was used to partition the brain of each participant into 226 cortical and subcortical areas. Subsequently, network connectivity was calculated within 10 RSNs as defined by previous fMRI studies [35, 36]. We also calculated network connectivity between all pairs of the 10 RSNs, as well as between each RSN and all other RSNs (i.e., one-versus-all-others). The detailed construction of the network is shown in Additional file 1.

### Statistical analysis

Comparisons between groups used one-way analysis of variance for continuous variables and chi-square tests for categorical variables. The significance level was set at p < 0.05. Post hoc analysis with least significance difference (LSD) correction (p < 0.05) was used to compare differences between two groups. All statistical analyses were conducted using SPSS v25 software (SPSS, Inc., Chicago, IL, USA).

To investigate correlations among polygenic scores (including GPS, GRS, RRS, GRS_n and RRS_n), lipid metabolites in the blood, CSF biomarkers, and cognitive performance in AD spectrum individuals, linear and binomial nonlinear regression analyses were employed, after controlling the covariates of age, gender, and years of education. The significance level was set at p < 0.05.

Each network metric (within-, one-versus-all-others-, and pairwise between-network connectivity) was compared across groups using generalized linear model analysis adjusted for age, gender, and education as covariates. All p values were adjusted for multiple comparisons (10 within-network metrics + 10 one-versus-all-others-network metrics + 45 pairwise between-network metrics = 65 comparisons) by controlling false discovery rate. Post hoc analysis was then performed to determine the significance of specific comparisons with network-based statistics (NBS) among groups (p < 0.01, FDR correction).

Additionally, the CCA was used to identify relationships between brain network connectivity measures and clinical phenotypes, CSF biomarkers, lipid related genetic variants, and lipoproteins in the serum of AD spectrum patients. Given a significant CCA mode, Pearson’s correlation was used to assess the correlation between the CCA mode and the corresponding set of original variables of which it consisted. Finally, the correlation coefficients were visualized using the radar plots in Fig. 5. Details on CCA were described in Additional file 1.

### Support vector machine classification

SVM was used in this study to classify AD spectrum in MATLAB based on a library (LIBSVM) [37]. The LIBSVM classifier algorithm was applied within Leave-one-out cross-validation (LOOCV). Grid search method and Gaussian radial basis function (RBF) kernels were used for parameter optimization. Post hoc analysis revealed nineteen lipoproteins and three gene scores were associated with network connectivity. Then, we performed Pearson correlation to find the functional connections which were correlated with all nineteen lipoproteins and all three gene scores. P values of correlation coefficient < 0.05 was considered statistically significant. Those functional connection were used in the classification by SVM. In order to quantify the performance of the final machine learning model, the accuracy, sensitivity, specificity, and area under the curve (AUC) were calculated to reduce the impact of deviations in the distribution of the training and testing sets. In addition, the accuracy (ACC) of testing set was assessed by permutation test with 1,000 epochs as described in previous studies [38].

### Path analysis

We further used SEM to examine the relationship among variables in radar plots (Additional file 1: Fig. S4). All variables in the radar plots were observed variables. Moreover, we constructed four variables (dyslipidemia, pathology, brain function, and cognition) as latent variables. Hypothesized relationships were constructed among variables based on the results of post hoc analysis. The causal path relationship of the 5 latent variables constituted the SEM structural model, and the relationship between latent variables and their corresponding observed variables constituted the SEM measurement model. SEM was conducted using IBM SPSS Amos version 22 statistical software (Amos Development Co., Armonk, NY, USA). For the hypothesized relationships, t-tests and path coefficients were determined using a bootstrapping approach with a sampling of 5000. The goodness of fit was assessed by chi square/degree of freedom ratio (CMIN/DF), root-mean-square error of approximation (RMSEA), goodness-of-fit index (GFI), adjusted GFI (AGFI), Tucker-Lewis Index (TLI), normed fit index (NFI), comparative fit index (CFI), and incremental (IFI). The significance level was set 0.05 in this study.

## Results

### Demographic, genetic, and molecular biomarkers, and neuropsychological data

There were no significant differences in age, gender, education, levels of thirty-eight serum lipid metabolites, or any candidate genotypes, except apolipoprotein E (APOE) genotypes within any groups of participants. Significant cognitive decline as signed by lower MMSE scores and higher ADAS-cog scores, gradually decreased Aβ level, and increased Tau and p-Tau levels were identified in ADS individuals compared to the CN. In terms of cognitive scores or CSF biomarkers, there were no significant differences between early MCI (EMCI) and late (LMCI) groups. No genotypes deviated from the Hardy–Weinberg equilibrium with all p values above 0.05. More details of demographic, lipid pathway-based genotypes, CSF biomarkers, and global cognition are displayed in Table 1 and Additional file 1: Tables S1, S2.

### Relationships among polygenic scores, lipid metabolites, CSF biomarkers and cognitive performance

**Fig. 1:**
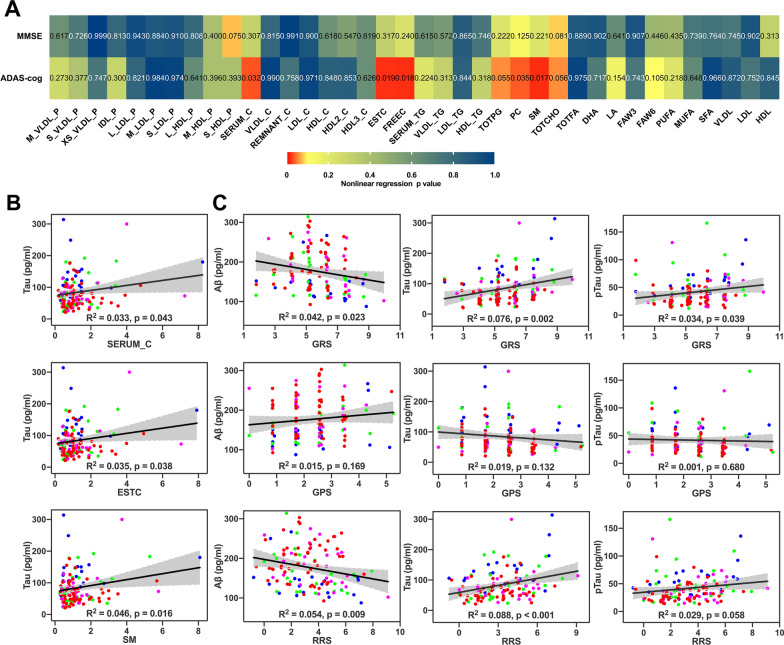
Regression analyses between polygenic scores, cerebrospinal fluid biomarkers, plasma cholesterol metabolites, and general cognition in the AD spectrum. **A** Nonlinear correlations were plotted in heat map between blood cholesterol metabolites and cognitive scores. The color bar indicated nonlinear regression p values ranging from 0 to 1. Two-tailed p values < 0.1 were considered significant. **B** Significant linear correlations were found between three cholesterol metabolism related markers in the blood and Tau level of cerebrospinal fluid in the AD spectrum. **C** Linear regression between polygenic scores and cerebrospinal fluid biomarkers revealed that GRS was significantly correlated with Aβ, Tau, and even p-Tau levels, in the contrast, GPS was not correlated with any of cerebrospinal fluid biomarkers; while relative risk scores (RRS = GRS—GPS) was significantly influenced the Aβ, Tau but not p-Tau levels in the AD spectrum. Grey bands indicated 95% confidence intervals and binomial nonlinear regressions were applied in Fig. 1B and 1C. Two-tailed p values < 0.1 were considered significant in Fig. 1B and 1C. The abbreviations of plasma cholesterol metabolites were are provided in Additional file 1: Table S2. *Aβ* amyloid 1 to 42 peptide, *Tau* total tau, *pTau* tau phosphorylated at the threonine 181 position, *MMSE* Mini-mental state examination, *ADAS-Cog* Alzheimer’s Disease Assessment Scale- Cognitive subscale, *GRS* genetic risk score including APOE, *GPS* genetic protective score, *RRS* relative risk score

First, binomial nonlinear connections were discovered between five cholesterol metabolism related biomarkers in the serum and ADAS-cog score, including serum total cholesterol (SERUM_C), esterified cholesterol (ESTC), free cholesterol (FREEC), phosphatidylcholine (PC), and sphingomyelins (SM). Besides, total phosphoglycerides (TOTPG), total choline (TOTCHO), and small HDL particles (S_HDL_P, including total lipids, phospholipids, total cholesterol, cholesterol esters, free cholesterol, and triglycerides) levels represented a correlation trend with ADAS-cog or MMSE scores. Then, three cholesterol metabolism related markers including SERUM_C, ESTC and SM were also linearly correlated with Tau but not Aβ and p-Tau levels of CSF in the ADS. In addition, linear regression between polygenic scores and CSF biomarkers disclosed that genetic risk scores (GRS) were significantly correlated with Aβ, Tau, and even p-Tau levels, while genetic protective score (GPS) was not correlated with any of CSF biomarkers. It is interesting that relative risk scores (RRS = GRS—GPS) was significantly influenced the Aβ, Tau but not p-Tau levels in the ADS. Of note, the correlations between GRS_n (GRS without APOE), RRS_n (RRS without APOE) and CSF markers were not found, so the graphs were not presented here. All the corresponding graphs above were plotted in Fig. 1. Meanwhile, regression analyses revealed that CSF biomarkers (including Aβ, Tau and p-Tau) could significantly impact global cognitive performance in a nonlinear manner (Additional file 1: Fig. S1).

### Dynamic trajectory of large-scale brain network roles across the AD spectrum

**Fig. 2:**
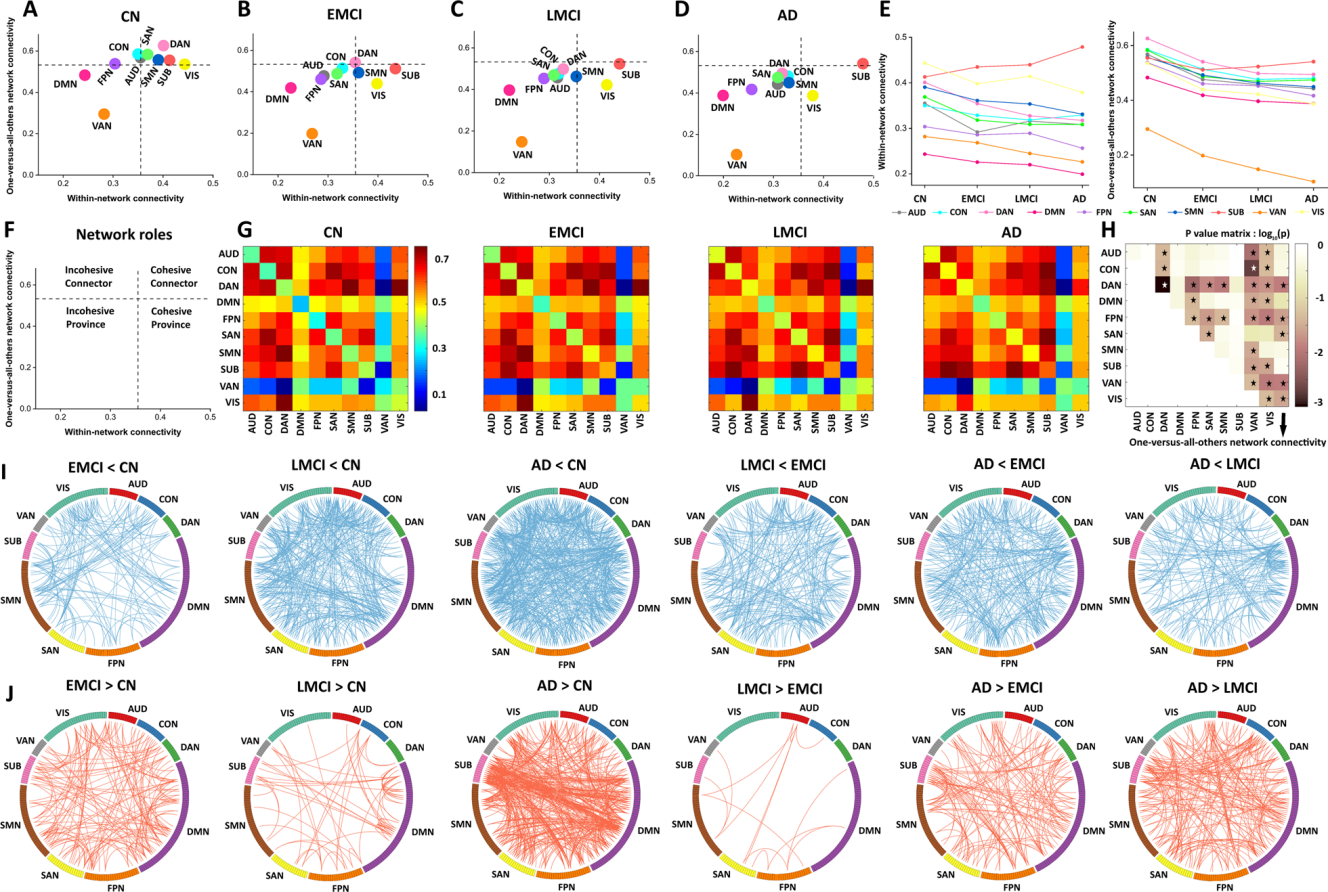
Network roles (F) in brain networks of CN (**A**), EMCI (**B**), LMCI (**C**), and AD (**D**); Dynamic trajectory of network role of large-scale RSNs within- and pairwise between-network connectivity matrices of the four groups (**G**); A Line chart (**H**) displays the dynamic trajectory of within- and one-versus-all-other network connectivity across the entire ADS; P value matrix of group differences in within-, one-versus-all-others-, and pairwise between-network connectivity in the ADS (**F**); Circos plot representation of significant group-level differences of neural connections among the ten RSNs in the ADS using the NBS method (I and J). Blue lines indicate decreased connectivity and red indicates increased connectivity in the ADS. *AUD* the auditory network, *CON* the cingulo-opercular network, *DAN* the dorsal attention network, *DMN* the default mode network, *FPN* the fronto-parietal network, *SAN* the salience network, *SMN* the sensory network, *SUB* the subcortical network, *VAN* the ventral attention network, *VIS* the visual network

To explore the dynamic trajectory both within- and between RSNs in ADS patients, pairwise functional connections (correlations) were extracted within- and between ten predefined large-scale functional brain networks: auditory network (AUD), cingulo-opercular network (CON), dorsal attention network (DAN), DMN, fronto-parietal network (FPN), SAN, sensory network (SMN), subcortical network (SUB), ventral attention network (VAN), and visual network (VIS), as derived from the brain atlas of Power et al*.* [35]. By mapping the group mean within-network connectivity (WNC) and between-network connectivity (BNC) to a 2D parameter space, the mean functional role of 10 RSNs was qualitatively described across the ADS (Fig. 2A–D). From the means of individual WNC and BNC values (depicted by horizontal and vertical dotted lines in Fig. 2F (detailed information provided in Additional file 1), the RSNs from the CN group were consequently classified into four network roles: cohesive connector (SAN, DAN, SMN, and SUB), cohesive provincial (VIS), incohesive connector (AUD, FPN and CON), or incohesive provincial (DMN and VAN) (Fig. 2A). In addition to DMN and VAN, which exhibited both weaker cohesive connector and cohesive provincial roles, the other eight networks in the ADS represented divergent network roles compared to those of the CN group. Specifically, SAN, DAN, and AUD represented incohesive provincial and connector networks in patients with EMCI, LMCI and AD, respectively, the converse of that observed in CN subjects (Fig. 2A–D). The graphs visually demonstrated how the network roles of these large-scale RSNs dynamically changed with severity of disease (Fig. 2E). Interestingly, the strengths of WNC and BNC exhibited a dynamically weakened trend, except for SUB, as disease progressed through the ADS, indicating that spatiotemporal patterns of large-scale RSNs represent a rebounding network mode rather than cascading network failure, as described previously [18].

### Group-level comparison of network connectivity in AD spectrum individuals

We next tested differences in WNC and BNC in terms of large-scale RSNs among the four groups. Firstly, we obtained distinctive WNC and BNC matrices of the 10 RSNs for the four groups (Fig. 2G). Clearly, five RSNs (DAN, FPN, SAN, VAN, and VIS) exhibited significantly differential WNC among the disease spectrum (Fig. 2H). Although VAN and DMN were found to be incohesive provincial networks in four groups (Fig. 2A–D), VAN exhibited significantly lower WNC and BNC across the ADS (Fig. 2A–D). Similarly, five RSNs, including DAN, SAN, SMN, CON, and AUD, were found to have incohesive connector roles and provincial networks that had lower connectivity in the ADS relative to CN subjects. It was noted that the FPN shifted from an incohesive connector to incohesive provincial network while the VIS changed from cohesive connector to a cohesive provincial network, both representing lower connectivity in the ADS relative to CN subjects. In addition, the SUB displayed more cohesive connector and cohesive provincial networking, having the greatest connectivity in ADS individuals compared with CN subjects. Furthermore, ADS patients also showed significantly differential one-versus-all-other-network connectivity in the DAN, FPN, SAN, VAN, and VIS networks compared with controls (Fig. 2H).

In addition, pairwise BNC was calculated as the mean connectivity between each pair of RSN. Connectivity profiles of patients with EMCI, LMCI and AD were compared with controls. Figure 2H demonstrates that pairwise BNC was significantly different for ADS patients in the following pairs: AUD-VAN, AUD-VIS, CON-DAN, CON-VAN, DMN-FPN, DMN-VAN, DAN-FPN, DAN-SMN, FPN-VAN, SUB-VIS, and VAN-VIS. Furthermore, post hoc analysis indicated that the source of the significant differences in these pairwise BNC groups was at the large-scale network level. Specifically, ADS patients were characterized by continuous hypoconnectivity and dynamically hyperconnected links among the ten predefined RSNs as disease progressed (Fig. 2I–J). These original alterations of large-scale networks may initially reproduce those spatiotemporal pattern discrepancies, accounting for proposed molecular pathophysiological mechanisms at the distributed network level.

### Correlation patterns of large-scale network connectivity with CSF biomarkers and cognitive performance in the AD spectrum

To explore the potential relationship between the dynamic trajectory of connectivity of the RSNs and CSF biomarkers or cognitive performance, a new method of combination network analysis and CCA was utilized. Recent studies have demonstrated that CCA, a powerful multivariate approach that seeks to identify clusters of maximal correlation between two groups of variables, can detect associations between structural or functional connectivity and other phenotypic measures [35, 39]. Using this method, we demonstrated that large-scale brain network abnormalities were significantly correlated with phenotypic variations and molecular biomarkers in the ADS. In the first step, univariate correlation was used to test the composition of the clinical CCA mode with each of the two clinical variables (MMSE and ADAS-cog). We observed that clinical CCA mode was highly correlated with MMSE score (r = 0.78, p < 0.0001) and ADAS-cog score (r = 0.78, p < 0.0001) (Additional file 1: Fig. S2A). Similarly, we identified that CSF CCA mode was highly correlated with levels of Tau (r = 0.72, p < 0.0001) and pTau (r = 0.68, p < 0.0001), and moderately correlated with Aβ42 (r = 0.55, p < 0.0001) (Additional file 1: Fig. S2B). As shown in Additional file 1: Fig. S2C, the network CCA mode was significantly correlated with 55 original network variables (Additional file 1: Table S4). In total, the CCA mode of network was significantly correlated with clinical variate CCA (Additional file 1: Fig. S2D, r = 0.93, p < 0.0001) and CSF CCA (Additional file 1: Fig. S2E, r = 0.95, p < 0.0001) modes, respectively.

### Correlation patterns of lipid pathway-based genetic variants and lipoproteins with large-scale network connectivity in AD spectrum patients

**Fig. 3:**
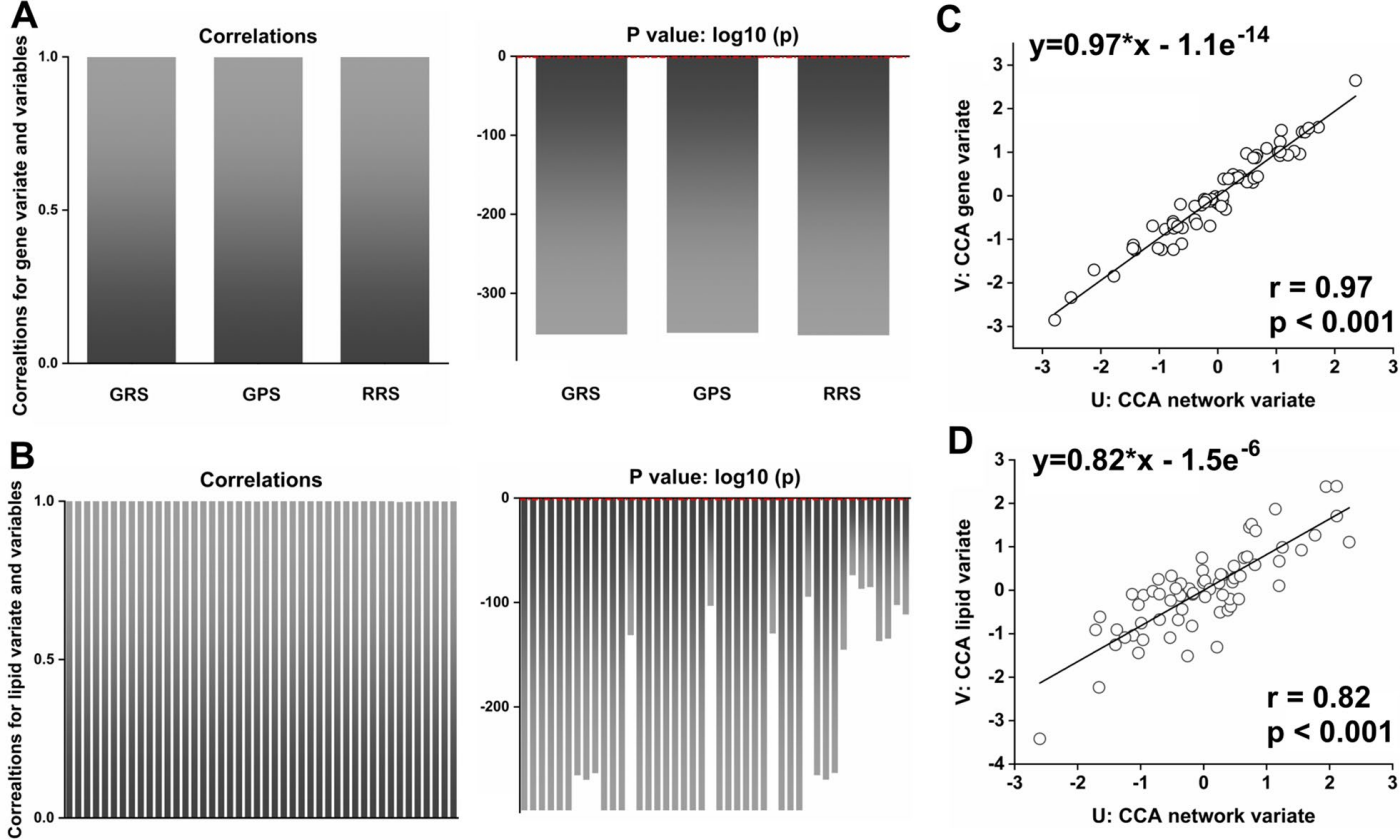
Correlations and their significance for patients with EMCI, LMCI or AD, for the following: **A** three gene score variables and gene CCA mode, **B** thirty-eight serum lipid variables and first level serum lipid CCA mode, **C** third pairs and **D** fourth pairs CCA mode. All data for lipid metabolites in blood were z-transformed. Note that P values in A and B were log10-transformed. Red dashed lines represent log10-transformed P values of 0.05. *GPS* genetic protective score, *GRS* genetic risk score, *RRS* relative risk score, *CCA* canonical correlation analysis

Similarly, we also performed CCA to ascertain the association of brain network connectivity measures with accumulated lipid-related genetic scores and lipoproteins in the blood of ADS patients. We firstly tested univariate correlations for each of the 3 gene variables and 38 serum lipid variables in order to better understand the composition of gene CCA and serum lipid CCA modes. We found that gene CCA mode was highly correlated with GRS (r = 1, p < 0.0001), GPS (r = 1, p < 0.0001) and RRS (r = 1, p < 0.0001) (Fig. 3A). As shown in Fig. 3B, serum lipid CCA mode was significantly correlated with all 38 original serum lipid variables (Additional file 1: Table S5). The results of third pair CCA mode of network and gene variate were again highly significant (Fig. 3C, r = 0.97, p < 0.0001), as was fourth pair CCA mode of network and serum lipid variable (Fig. 3D, r = 0.82, p < 0.0001).

In order to determine the potential for APOE ε4 genotype to alter the association between lipid metabolism-related genes and dynamic changes in RSNs, we constructed a second gene CCA mode and found that second order gene CCA mode was highly correlated with GRS_n (r = 1, P < 0.0001) and RRS_n scores (r = 1, P < 0.0001) after removing the APOE ε4 genotype (Additional file 1: Fig. S3A). Fifth pair CCA mode of the network and three gene score variables where removed APOE ε4 OR values were also significantly correlated (Additional file 1: Fig. S3C, r = 0.94, p < 0.0001).

### Post hoc analysis revealed the potential of distinctive lipid-related genetic scores and lipoproteins on large-scale network connectivity, CSF biomarkers, and cognitive performance

To determine the direction and magnitude of these associations between network CCA mode and a single variate of a clinical indicator, we conducted post hoc correlation analysis. As illustrated in Additional file 1: Fig. S4A, nineteen lipoproteins were mostly associated with increased network connectivity and seven with decreased network connectivity within- and between- ten predefined RSNs. Furthermore, GRS was positively associated with increased network connectivity within the SUB and negatively associated with altered network connectivity between CON-VAN, DAN-VAN, FPN-VAN, SAN-VAN, SUB-VAN, VAN-VIS, and AUD-VAN, while GPS was negatively associated with decreased network connectivity between the FPN and SUB. Similarly, RRS was positively associated with decreased network connectivity between DMN-SUB, DAN-SUB, FPN-SUB, CON-DMN, AUD-CON, and negatively associated with decreased network connectivity between AUD-VAN, and DAN-VAN, whereas RRS was associated with increased network connectivity within the SUB. In addition, MMSE was negatively correlated with decreased network connectivity between DMN and SAN, while ADAS-cog and Tau were mostly associated with increased network connectivity between SAN-SUB, and DMN-SAN, as shown in Additional file 1: Fig. S4B. It is noteworthy that GRS was only associated with decreased network connectivity between SAN-VAN after removing the effects of the APOE ε4 genotype (Additional file 1: Fig. S5). Detailed information for these correlation coefficients ® and p values are described in Additional file 1: Table S6.

### SVM analyses identified potential lipid-associated imaging biomarker for AD spectrum

**Fig. 4:**
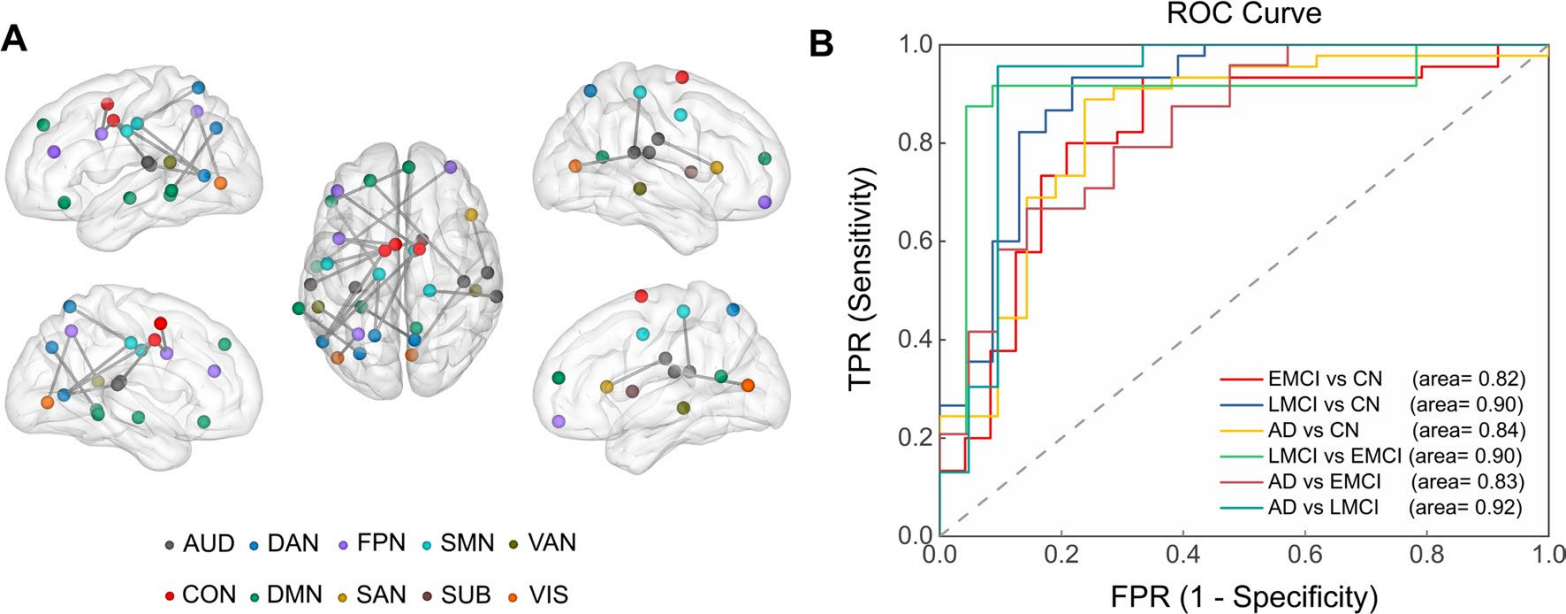
Lipid-associated imaging biomarker for classifying AD spectrum. **A** Twenty-two functional connections were used for classification between groups. Node colors represent Power-atlas cortical and subcortical regions consisting of ten RSNs. **B** Functional connections that showed group-level differences were used as the inputs for binary classification. All p values of area under curve were < 0.001. *AUD* the auditory network, *CON* the cingulo-opercular network, *DAN* the dorsal attention network, *DMN* the default mode network, *FPN* the fronto-parietal network, *SAN* the salience network, *SMN* the sensory network, *SUB* the subcortical network, *VAN* the ventral attention network, *VIS* the visual network, *ROC* receiver operating characteristic, *TPR* true positive rate, *FPR* false positive rate, *CN* cognitively normal, *EMCI* early mild cognitive impairment, *LMCI* late mild cognitive impairment, *AD* Alzheimer’s disease

After post-hoc analysis, we performed correlation analysis and found that there were six functional connections significantly correlated to all nineteen lipoproteins and sixteen functional connections related to all three gene scores (Additional file 1: Table S7). Then, these twenty-two features were used for classification. The SVM model revealed that the lipid-associated twenty-two functional connections represented higher capacity to discriminate disease spectrum (AUC between 0.82 and 0.92), as shown in Fig. 4.

### Path analysis

**Fig. 5:**
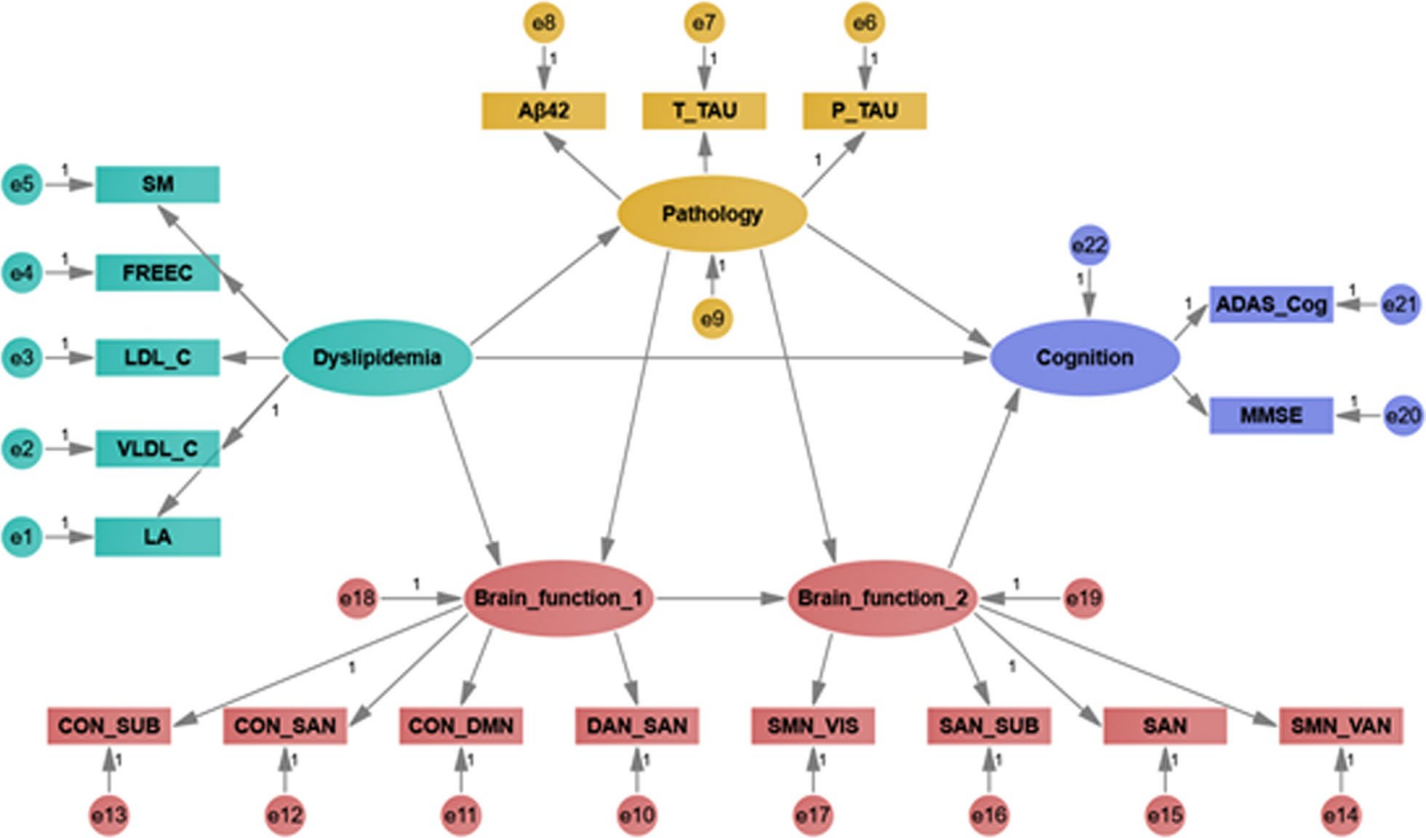
Structural equation model for direct, indirect and mediation relationship. Ellipses: latent variables; boxes: observed variables. *CON* the cingulo-opercular network, *DAN* the dorsal attention network, *DMN* the default mode network, *SAN* the salience network, *SMN* the sensory network, *SUB* the subcortical network; *VAN* the ventral attention network, *VIS* the visual network, *Aβ* amyloid-1 to 42 peptides, *T_Tau* total tau, *P_Tau* tau phosphorylated at the threonine 181 position, *MMSE* mini-mental state examination, *ADAS-Cog* Alzheimer’s Disease Assessment Scale-Cognitive Subscale. The abbreviations of plasma cholesterol metabolites were are provided in Additional file 1: Table S2

First, when all the significant measurement variables in the post-hoc analysis results were included in the model, the fitting results were suboptimal. Then, we drop some observed variables from the final SEM model based on the fit of parameters and the modification indices. All fit indices of the final model indicate excellent fit to the model (see Additional file 1: Table S8). Figure 5 and Additional file 1: Table S9 showed the results of testing the structural model. From the analysis, we found that dyslipidemia (β = 0.31, p = 0.02) produced significant effect on brain function 1, which was positively association with brain function 2 (β = 0.96, p < 0.001), indicating dyslipidemia may induce brain networks reorganization at the large-scale levels. In addition, CSF biomarkers had a positive and significant influence on cognition (β = 0.41, p < 0.001).

## Discussion

This is the first study focusing on the potential of lipid-related genes and proteins to influence the dynamic trajectory of large-scale RSNs, CSF biomarkers, and cognitive decline in ADS patients using a CCA approach. The present study shed mechanistic light on the role of lipid metabolites disturbance in promoting large-scale RSNs disruption and accelerating CSF biomarker deposition and subsequently caused cognitive decline in ADS individuals. These findings provided novel insight for uncovering the neural link between lipid metabolites and cognitive decline at a large-scale network level and expanding our understanding of the mechanisms underlying AD pathophysiology.

Although it is not well-established that potential relationships between lipid metabolites and AD exist, the majority of studies have reported that abnormal lipid metabolites apparently increased the risk of cognitive decline and substantially contribute to the development and progression of AD [3, 7, 40, 41]. Recently, a meta-analysis reported that high midlife total serum cholesterol significantly increases the risk of late-life AD, and may correlate with the onset of AD pathology [42]. A prospective study with a large-cohort sample in which 22,623 participants were recruited established that the concentration of cholesterol esters relative to total lipids in large HDL and the total cholesterol to total lipid ratio in very large VLDL significantly increased the risk of incidence of dementia [7]. We also found that lipid metabolites, including genes and lipoproteins were markedly associated with CSF biomarkers and cognitive impairment, also supporting the hypothesis that lipid metabolic dysfunction substantially contributes to AD pathophysiology via interference through progressive neuropathological changes of CSF biomarkers and declining cognitive function across the entire ADS.

Disrupted network integrity, including abnormal structural and functional network connectivity, was preferentially targeted by specific genetic variants or molecular pathology in preclinical AD, or mapped the clinical phenotype with disease progression and supported the recent description of the theoretical framework and empirical evidence of AD [24, 43]. As such, brain network integrity emerged as potential intermediate biomarkers to bridge upstream determinants (gene and molecular pathology) and downstream effects (clinical phenotypes) [23, 24]. However, the complicated association that the dynamic spatiotemporal patterns of brain network integrity linking molecular pathology and cognitive decline in ADS individuals remains largely unclear. According to cascading network failure theory, distinct DMN subsystems representing differential spatiotemporal evolution correspond with the AD pathophysiological response, and differentially affected by AD pathological biomarkers including Aβ deposition and tau tangles, subsequently leading to stereotypic network-based cognitive decline in ADS patients [18, 22]. This study firstly described the progressive changes in spatiotemporal network patterns within the DMN system in ADS patients. We then further identified changes in dynamic trajectory within- and between networks reflected by the active capability of network inner cohesion and connectors beyond the DMN across the entire ADS. More attention should be focused on whether such changes in dynamic trajectory in large-scale RSNs are cascading failure or not. In contrast, a proportion of the networks represented enhanced network inner cohesion or exhibited network connector roles as the disease progressed. Compelling evidence has been reported that a gradual decrease in connectivity of RSNs is associated with amyloid deposition that accelerates disease progression, while the commonly observed increase in connectivity of RSNs also found in preclinical and prodromal AD patients has been interpreted as a compensatory phenomenon supporting better performance on cognitive tasks [44, 45]. However, this enhanced network connectivity is the consequence of transient compensation to network disruption or an adaptive response to AD pathophysiological processes that still require identification through additional study.

Importantly, the dynamic changes in large-scale networks over the course of disease that were also observed were significantly affected by lipid-related genetic variants and lipoproteins, CSF biomarkers, and cognitive function, confirming the biological nature of the predictable correlation with network connectivity by linking upstream molecular pathology and downstream clinical phenotype to the preclinical stage of AD. Furthermore, post hoc analysis was performed to trace the source of these system-level correlations and identified that distinctive connectivity within- and between networks was specifically related to the effects of accumulated lipid-related genetic variants or lipoproteins, neuropathological biomarkers, in addition to cognitive decline. Due to changes in lipids often prior to molecular pathology and cognitive decline, we hypothesized that compromised large-scale networks and CSF biomarkers may mediate the effects of lipid metabolites on cognitive decline with progression of AD. Previously, structural atrophy or functional decoupling of RSNs were, at least partly, ascribed to abnormalities in lipid metabolites which suggested that lipid metabolites may be a vulnerable molecular substrate of large-scale RSNs [28, 34]. More importantly, disturbed lipid metabolites and dynamic brain network changes occurred prior to measurable amyloid deposition and tau tangles related to ageing [18], while lipid pathway genetic variants, including APOEε4 genotype and lipoproteins markedly enhanced the disruption of brain network architecture in preclinical AD patients [34, 46] and even in the cognitively normal elderly [47, 48]. In addition, carriers of the APOEε4 allele, the strongest risk factor for sporadic late-onset AD, represented a specific phenotype in which the relationship with brain networks preceded any measurable systems or molecular level changes in cognitively normal subjects [49–51]. Furthermore, cholesterol-related genetic risk scores were associated with hypometabolism in AD-affected brain regions, even when controlling for the effects of APOE ε4 gene dose [52].

In this regard, we putatively identified that the dynamic trajectory changes of large-scale networks observed in this study may be induced because of a lipid-driven pathological interaction with Aβ abnormal deposition and tau-related neurofibrillary tangles and then promoted cognitive decline to dementia. From the path analysis, we found that dyslipidemia directly influenced brain function network reorganization leading to cognitive impairment or indirectly affected the CSF biomarkers levels and subsequently caused cognitive decline.

Another interesting finding of the present study was the SVM classifier model. This SVM classifier achieved a relatively high performance and implies that a significantly important role of lipid metabolism in the onset and neuropathology of AD. Lipid associated neuroimaging biomarkers would serve as a good potential biomarker for ADS diagnoses and an invention target to early prevent AD incidence.

Several limitations should be noted. Firstly, the 38 lipid-related genetic variants and lipoproteins selected in this multimodal cross-sectional study may underestimate the potential of lipid metabolites for the early detection and diagnosis of AD. Lipidomic approaches should be considered in order to explore the pathogenesis of AD, because this provides a new tool to investigate the association between blood-based genetic variants or changes in lipoproteins in the serum or plasma and the pathological mechanisms of CNS disorders. Secondly, longitudinal studies should be performed to explore the potential biomarkers of lipid metabolites in AD pathophysiology, validate the neural links between changes in lipids and neuropathology, and determine the causal contributions of lipid metabolite disturbance and disrupted network integrity, in addition to cognitive decline in ADS patients.

## Conclusions

To sum, we demonstrated that abnormal lipid metabolite changes induced the disruption of large-scale RSNs and CSF biomarker deposition, which then promoted cognitive impairment in preclinical and clinical AD. In addition, we also found that dynamic trajectory of large-scale RSNs represented a rebounding mode rather than a cascading failure mode with disease progression in the ADS. These findings provided new evidence in which an effective strategy for early prevention or disease-modifying therapy that targets the metabolites of lipid-related genetic variants or lipoproteins for late-onset AD, which would significantly improve our understanding of the mechanisms underlying the association of lipid molecules and AD pathophysiology.

## Supplementary Information

**Additional file 1**: **Table. S1** Summary of multiple SNPs based on GWAS and large-scale meta-analyses for lipid pathway-based genotypes. **Table. S2** Concentrations of cholesterol metabolites in blood correlated with cognition across the AD spectrum. **Table. S3** Anatomical locations of the 226 regions of interest used to characterize the 10 resting-state networks. **Table. S4** Canonical correlation coefficients and p values within- and between the ten predefined RSNs. **Table. S5** Canonical correlation coefficients and p values of lipid-related genetic scores and lipoproteins. **Table. S6** Post hoc analysis revealed correlation coefficients and p values among lipid-related genetic scores and lipoproteins, large-scale network connectivity, CSF biomarkers, and cognitive performance. **Table. S7** Twenty-two functional connection links used for classification between groups. **Table. S8** Modified model fit indices. Table S9 Results of Structural Path model of direct effects. **Figure. S1** Nonlinear curves fitted between cerebrospinal fluid biomarkers and global cognitive performance. Abbreviations: MMSE, mini-mental state examination; ADAS-cog, Alzheimer’s disease assessment scale-cognitive section; Aβ, amyloid 1 to 42 peptide; Tau, total tau; pTau, tau phosphorylated at the threonine 181 position; CSF, cerebrospinal fluid. **Figure. S2** Correlations and their significance between the following in patients with EMCI, LMCI and AD: the two clinical cognitive performance variables and clinical CCA mode (A); the three cerebrospinal fluid biomarker variables and CSF CCA mode (B); fifty-five within and pairwise between-network variables and network CCA mode (C); first pairwise CCA mode (D), and second pairwise CCA mode (E). Note P values in A, B and C, have been log10-transformed. Red dashed lines represent a log10-transformed P value of 0.05. Abbreviations: MMSE, mini-mental state examination; ADAS-cog, Alzheimer’s disease assessment scale-cognitive section; Aβ, amyloid 1 to 42 peptide; Tau, total tau; pTau, tau phosphorylated at the threonine 181 position; CSF, cerebrospinal fluid; CCA, canonical correlation analysis. **Figure. S3** Correlations and their significance between the following in patients with EMCI, LMCI and AD: the three gene score variables and the second gene CCA mode (A), the thirty-eight serum lipid variables and serum lipid CCA mode (B), the fifth pairs of CCA modes (C) and sixth pairs of CCA modes (D). All data for cholesterol metabolites in the blood were z-transformed. Note that the P values in A and B were log10-transformed. Red dashed lines represent a log10-transformed P value of 0.05. Abbreviations: GPS, genetic protective score; GRS_n, genetic risk score without APOE ε4; RRS, relative risk score without APOE ε4; CCA, canonical correlation analysis. **Figure. S4** Radar plots indicating patterns of association for serum lipid, cognitive performance, CSF biomarkers, and polygenic scores to network connectivity. Values displayed by the dots in the radar plots are values of Pearson’s correlation coefficients. All nodes represent a statistically significant correlation coefficient (p value<0.05). Abbreviations: AUD, the auditory network; CON, the cingulo-opercular network; DAN, the dorsal attention network; DMN, the default mode network; FPN, the fronto-parietal network; SAN, the salience network; SMN, the sensory network; SUB, the subcortical network; VAN, the ventral attention network; VIS, the visual network; LDL, low density lipoprotein; MMSE, mini-mental state examination; ADAS-cog, Alzheimer’s disease assessment scale-cognitive section; Tau, total tau; GPS, genetic protective score; GRS, genetic risk score; RRS, relative risk score. The abbreviations of plasma cholesterol metabolites were are provided. **Figure. S5** Radar plots demonstrating patterns of association of cognitive performance, CSF biomarkers, and gene scores to network connectivity. Values displayed by the dots in the radar plots are values of Pearson’s correlation coefficients. All nodes represent a statistically significant correlation coefficient (p value<0.05). Abbreviations: AUD, the auditory network; DAN, the dorsal attention network; FPN, the fronto-parietal network; SAN, the salience network; SUB, the subcortical network; VAN, the ventral attention network; MMSE, mini-mental state examination; ADAS-cog, Alzheimer’s disease assessment scale-cognitive section; Tau, total tau; GPS, genetic protective score; GRS_n, genetic risk score without APOE ε4.

## Data Availability

The datasets generated and/or analyzed during the current study are available in the Alzheimer’s disease Neuroimaging Initiative repository, http://www.loni.ucla.edu/ADNI.

## Abbreviations

RSNs: Resting-state networks
AUD: The auditory network
CON: The cingulo-opercular network
DAN: The dorsal attention network
DMN: The default mode network
FPN: The fronto-parietal network
SAN: The salience network
SMN: The sensory network
SUB: The subcortical network
VAN: The ventral attention network
VIS: The visual network
CMIN/DF: Chi square/degree of freedom ratio
RMSEA: Root-mean-square error of approximation
GFI: Goodness-of-fit index
AGFI: Adjusted GFI
TLI: Tucker-Lewis index
NFI: Normed fit index
CFI: Comparative fit index
IFI: Incremental fit index
MMSE: Mini-mental state examination
ADAS-cog: Alzheimer’s disease assessment scale-cognitive section
Aβ: Amyloid 1 to 42 peptide
Tau: Total tau
pTau: Tau phosphorylated at the threonine 181 position
CSF: Cerebrospinal fluid
MMSE: Mini-mental state examination
ADAS-cog: Alzheimer’s disease assessment scale-cognitive section
Aβ: Amyloid 1 to 42 peptide
Tau: Total tau
pTau: Tau phosphorylated at the threonine 181 position
CSF: Cerebrospinal fluid
CCA: Canonical correlation analysis
GPS: Genetic protective score
GRS_n: Genetic risk score without APOE ε4
RRS: Relative risk score without APOE ε4
