# Supplementary material for: Dyslipidemia induced large-scale network connectivity abnormality facilitates cognitive decline in the Alzheimer’s disease
Source: J Transl Med. 2022 Dec 6;20:567. doi: 10.1186/s12967-022-03786-w (PMC9724298; doi:10.1186/s12967-022-03786-w)
Supplement: Supplementary file 1 — Additional file 1: Table. S1 Summary of multiple SNPs based on GWAS and large-scale meta-analyses for lipid pathway-based genotypes. Table. S2 Concentrations of cholesterol metabolites in blood correlated with cognition across the AD spectrum. Table. S3 Anatomical locations of the 226 regions of interest used to characterize the 10 resting-state networks. Table. S4 Canonical correlation coefficients and p values within- and between the ten predefined RSNs. Table. S5 Canonical correlation coefficients and p values of lipid-related genetic scores and lipoproteins. Table. S6 Post hoc analysis revealed correlation coefficients and p values among lipid-related genetic scores and lipoproteins, large-scale network connectivity, CSF biomarkers, and cognitive performance. Table. S7 Twenty-two functional connection links used for classification between groups. Table. S8 Modified model fit indices. Table S9 Results of Structural Path model of direct effects. Figure. S1 Nonlinear curves fitted between cerebrospinal fluid biomarkers and global cognitive performance. Abbreviations: MMSE, mini-mental state examination; ADAS-cog, Alzheimer’s disease assessment scale-cognitive section; Aβ, amyloid 1 to 42 peptide; Tau, total tau; pTau, tau phosphorylated at the threonine 181 position; CSF, cerebrospinal fluid. Figure. S2 Correlations and their significance between the following in patients with EMCI, LMCI and AD: the two clinical cognitive performance variables and clinical CCA mode (A); the three cerebrospinal fluid biomarker variables and CSF CCA mode (B); fifty-five within and pairwise between-network variables and network CCA mode (C); first pairwise CCA mode (D), and second pairwise CCA mode (E). Note P values in A, B and C, have been log10-transformed. Red dashed lines represent a log10-transformed P value of 0.05. Abbreviations: MMSE, mini-mental state examination; ADAS-cog, Alzheimer’s disease assessment scale-cognitive section; Aβ, amyloid 1 to 42 peptide; Tau, total tau; p [file 12967_2022_3786_MOESM1_ESM.docx]

**Dyslipidemia induced large-scale network connectivity abnormality facilitates cognitive decline in the Alzheimer’s disease**

Supplementary material contains methods and the complete results for the lipid data selection, CCA analysis and post hoc analysis. Some tables are the results of network node coordinate and path analysis.

**Supplementary Text**

**Methods**

**Inclusion and exclusion criteria**

Firstly, CN subjects were considered free of memory complaints. Mild cognitive impairment (MCI) patients were categorized into EMCI or LMCI subgroups based on education adjusted scores on the delayed recall of one paragraph from the Wechsler Memory Scale Logical Memory II: (1) EMCI: ≥16 years: 9-11; 8-15 years: 5-9; 0-7 years: 3-6; (2) LMCI: ≥16years: ≤8; 8-15 years: ≤4; 0-7 years: ≤2. Inclusion criteria for mild AD subjects were as follows: mini-mental state examination (MMSE) scores between 20-26, clinical dementia rating scale of 0.5 or 1.0. More detailed information regarding the inclusion and exclusion criteria for all subjects is available within the ADNI-2 protocol document (http://adni.loni.usc.edu/wp-content/themes/freshnews-dev-v2/documents/clinical/ADNI-2_Protocol.pdf).

**Selection and construction of plasma lipids**

The Alzheimer’s Disease Metabolomics Consortium (ADMC) has enabled the plasma metabolic data available from the Nightingale Platform NMR Analysis of Lipoproteins and Metabolites dataset. After excluding incomplete or missing information of lipids (n=68) and ratio values (n=65), 95 out of 228 lipid indexes were used for the above analysis.

First, for those macromolecules, such as medium very low-density lipoprotein particles (M_VLDL_P), which was comprised of seven micromolecular total lipids, phospholipids, total cholesterol, cholesterol esters, free cholesterol, and triglycerides. For statistical convivence, we sum up these small molecules to construct a composite concentration of this lipid macromolecule. This computation strategy was also applied for the other nine macromolecules, including small VLDL particles (S_VLDL_P), very small VLDL particles (XS_VLDL_P), intermediate density lipoprotein particles (IDL_P), large low density lipoprotein particles (L_LDL_P), medium LDL particles (M_LDL_P), small LDL particles (S_LDL_P), large high density lipoprotein particles (L_HDL_P), medium HDL particles (M_HDL_P), and small HDL particles (S_HDL_P). Then we constructed 35 plasma lipid indexes (35=95–7x10+10). Then all 35 lipids data were standardized according to the formula of z values = (x-μ)/σ, where x was the data value, μ was the mean value, and σ the standard deviation. For calculated simplicity of multiple indexes, z values were transformed using an inverse logarithm algorithm with a base of 2. Notably, considering the opposite effects of HDL compared with LDL or VLDL, HDL correlated data were further converted into their reciprocal form. In addition, all levels of indicators containing HDL, LDL, VLDL were summarized to construct an overall HDL, LDL, VLDL level, respectively. Eventually, thirty-eight lipid metabolic biomarkers were obtained (**Supplementary Table 2**).

**Resting-state fMRI data parameter and preprocessing**

Resting-state functional magnetic resonance imaging (rs-fMRI) images at baseline were collected from ADNI-GO, ADNI-2 and ADNI-3 projects. All subjects were scanned using a Philips Healthcare scanner with a 3.0 tesla field strength. Sequence parameters were as follows: echo time = 30ms, repetition time = 3000ms, flip angle = 80°, matrix = 64 × 64, pixel space = 3.3 × 3.3 × 3.3 mm^3^, number of slices = 48, slice thickness = 3.3 mm, and time points = 140.

All rs-fMRI images were pre-processed using SPM12 software (<http://www.fil.ion.ucl.ac.uk/spm/download/spm12>) and the RESTplus V1.2 toolkit (<http://www.restfmri.net/forum/RESTplusV1.2>) in MATLAB 2012b (MathWorks, Inc., Natick, MA, USA) in accordance with the following steps: the first ten volumes were deleted to ensure the signal was equilibrated, and slice timing correction was conducted to correct acquisition time differences among slices. Head motion correction was performed using a six-parameter rigid-body transformation method to exclude subjects with head motion exceeding 2 mm translation or 2° rotation. Friston 24-dimensional head motion regressors were used, to largely minimize the effects of possible head movement. Realigned images were then further spatially normalized to the standard template of the Montreal Neurological Institute (MNI) and resampled to a 3 × 3 × 3 mm^3^ voxel size. Finally, normalized images were smoothed using a Gaussian kernel of 6 × 6 × 6 mm^3^.

**Functional connectivity within- and between RSNs**

The atlas of Power *et al.*[1] was used to partition the brain of each participant into 264 cortical and subcortical areas. In the present study, 226 out of the 264 regions of interest (ROIs) were assigned to 10 well-established large-scale resting-state networks (RSNs)[1-3], including the auditory network (AUD), cingulo-opercular task control network (CON), dorsal and ventral attention networks (DAN and VAN), default mode network (DMN), fronto-parietal task control network (FPN), salience network (SAN), sensorimotor network (SMN), subcortical network (SUB), and visual network (VIS). Detailed information for the ten predefined networks is illustrated in **Supplementary Table 3**. In addition, the functional connectivity between all pairs of the 226 ROIs was estimated for each subject using wavelet coherence[4], resulting in a 226×226 connectivity matrix for each participant.

Subsequently, three types of network connectivity for the 10 RSNs were computed from the connectivity matrices: within-network, one-versus-all-others-network, and pairwise network connectivity between two ROIs, as follows:


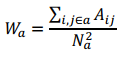
Firstly, as defined in Gu *et al.*[5], within-network connectivity (W𝑎) for each RSN (𝑎 ∈{1,2, ⋯,10} ) was calculated as the mean connectivity across all links within the RSN normalized by the square of the number of nodes (ROIs) within the network 𝑎, as follows:

where A _i j_ was the 226×226 connectivity matrix; N𝑎 denotes the number of nodes within the network 𝑎; and 𝑖 and 𝑗 denote powers of ROIs.

Secondly, for a network with N nodes (here, N = 226), one-versus-all-others-network connectivity (B𝑎−𝜀) was computed as the mean connectivity across all the links from one reference RSN (𝑎 ∈ {1,2, ⋯,10}) to all other RSNs (𝜀 ∈ {1,2, ⋯,10} − 𝑎) normalized by the product of the number of nodes
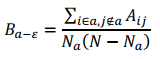
within the reference network 𝑎 and all other networks, as follows:


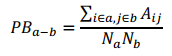
Thirdly, pairwise connectivity between networks (PB𝑎-𝑏) was computed as the mean connectivity across all the links between two RSNs, 𝑎 and 𝑏 (𝑎, 𝑏 ∈ {1,2, ⋯,10}; 𝑎 ≠ 𝑏), normalized by the product of the number of nodes within the two networks, as follows:

**Canonical correlation analysis (CCA)**

To explore the complex relationship between lipid pathway-based genetic variants and lipoproteins, and the dynamic trajectory of RSN connectivity, CSF biomarkers, in addition to cognitive performance, the canonical correlation analysis (CCA) was used to link brain network connectivity measures with 2 clinical phenotypes, CSF biomarkers, and lipid related genetic variants and lipoproteins in the serum of AD spectrum patients. Recent studies have shown that CCA, a powerful multivariate approach that seeks to identify clusters of maximal correlation between two groups of variables, can detect associations between structural or functional connectivity and other phenotypic measures [1, 6]. In the present study, CCA was used to link clinical, CSF and gene expression data with RSN connectivity in patients with EMCI, LMCI, and AD. There were six sets of variables, including a set of variables including within- and pairwise between-network connectivity, one consisting of each patient’s clinical cognitive performance, another that included Aβ, Tau and pTau concentration, a set consisting of each patient’s serum lipid indicators, a set consisting of each patient’s GRS, GPS and RRS score with APOE ε4 and a set consisting of each patient’s GRS, GPS and RRS score without APOE ε4. CCA provided a set of modes that maximally correlated network, clinical, CSF, serum lipid and gene variables. For each CCA mode, a permutation testing procedure was used to test the significance of the corresponding canonical correlation [7, 8]. P values for the correlation of each CCA mode pair were explicitly corrected for multiple testing across all CCA mode pairs estimated[8]. A CCA mode pair was considered significantly correlated only if both tests rejected the null hypothesis of no association at the level of P < 0.05. Given a significant CCA mode, Pearson’s correlation between the CCA mode and the corresponding set of original variables of which it consisted was then assessed. We also computed Pearson’s correlation between the value of the data in clinical, CSF, serum lipid and gene variables with the brain network CCA mode. Finally, the correlation coefficients were visualized using radar plots**.**

**References**

1. Power JD, Cohen AL, Nelson SM, Wig GS, Barnes KA, Church JA, Vogel AC, Laumann TO, Miezin FM, Schlaggar BL, Petersen SE: **Functional network organization of the human brain.** *Neuron* 2011, **72:**665-678.<https://doi.org/10.1016/j.neuron.2011.09.006>

2. Cole MW, Reynolds JR, Power JD, Repovs G, Anticevic A, Braver TS: **Multi-task connectivity reveals flexible hubs for adaptive task control.** *Nat Neurosci* 2013, **16:**1348-1355.<https://doi.org/10.1038/nn.3470>

3. Mohr H, Wolfensteller U, Betzel RF, Misic B, Sporns O, Richiardi J, Ruge H: **Integration and segregation of large-scale brain networks during short-term task automatization.** *Nat Commun* 2016, **7:**13217.<https://doi.org/10.1038/ncomms13217>

4. Chen G, Ward D, Xie C, Li W, Wu Z, Jones JL, Franczak M, Antuono P, Li S-J: **Classification of Alzheimer disease, mild cognitive impairment, and normal cognitive status with large-scale network analysis based on resting-state functional MR imaging.** *Radiology* 2011, **259:**213–221.<https://doi.org/10.1148/radiol.10100734/-/DC1>

5. Latora V, Marchiori M: **Efficient behavior of small-world networks.** *Phys Rev Lett* 2001, **87:**198701.<https://doi.org/10.1103/PhysRevLett.87.198701>

6. Smith SM, Nichols TE, Vidaurre D, Winkler AM, Behrens TE, Glasser MF, Ugurbil K, Barch DM, Van Essen DC, Miller KL: **A positive-negative mode of population covariation links brain connectivity, demographics and behavior.** *Nat Neurosci* 2015, **18:**1565-1567.<https://doi.org/10.1038/nn.4125>

7. Dosenbach NU, Fair DA, Cohen AL, Schlaggar BL, Petersen SE: **A dual-networks architecture of top-down control.** *Trends Cogn Sci* 2008, **12:**99-105.<https://doi.org/10.1016/j.tics.2008.01.001>

8. Fair DA, Dosenbach NUF, Church JA, Cohen AL, Brahmbhatt S, Miezin FM, Barch DM, Raichle ME, PetersenB SE, Schlaggar rL: **Development of distinct control networks through segregation and integration.** *Proceedings of the National Academy of Sciences of the United States of America* 2007, **104:**13507–13512.

**Additional file 1: Table S1 Summary of multiple SNPs based on GWAS and large-scale meta-analyses for lipid pathway-based genotypes.**

| **SNP** | **Chromosome** | **Closest gene** | **Protective/Risk allele** | **OR** | **Reference** |
| --- | --- | --- | --- | --- | --- |
| rs11136000 | 8 | CLU | T | 0.86 | Harold D, 2009 |
| rs5930 | 19 | LDLR | A | 0.85 | Olgiati P, 2011 |
| rs1799986 | 12 | LRP1 | T | 0.92 | Wang Y, 2017 |
| rs3851179 | 11 | PICALM | A | 0.85 | Harold D, 2009 |
| rs2070045 | 11 | SORL1 | G | 1.13 | Wang Z, 2016 |
| rs5882 | 16 | CETP | A | 1.11 | Chen J, 2014 |
| rs2230808 | 9 | ABCA1 | G | 1.10 | Wollmer M, 2010 |
| rs744373 | 2 | BIN1 | C | 1.17 | Hollingworth p, 2011 |
| rs429358 | 19 | APOE | - | - | Xiao E, 2017 |
| rs7412 |  |  | - | - |  |

**Note:** Genetic risk score of APOE was calculated based on odds ratios for ε2, ε3, and ε4 alleles after haplotypes were determined based on the combined genotypes of rs429358 and rs7412 published in Xiao *et al*., with OR values defined as: ε2/ε2 OR = 0.000, ε2/ε3 OR = 0.322, ε3/ε3 OR = 0.693, ε2/ε4 OR = 1.493, ε3/ε4 OR = 1.914, and ε4/ε4 OR = 3.252. **Abbreviations**: SNP, single nucleotide polymorphism; GWAS, genome-wide association study; OR, odds ratio for the minor allele based on literatures; CLU, clusterin; LDLR, low density lipoprotein receptor; LRP1, low density lipoprotein receptor-related protein 1; PICALM, phosphatidylinositol-binding clathrin assembly protein; SORL1, sortilin-related receptor 1; CETP, cholesterol ester transfer protein; ABCA1, ATP-binding cassette transporter A1; BIN1, bridging integrator 1; APOE, apolipoprotein E.

**Additional file 1: Table S2 Concentrations of cholesterol metabolites in blood correlated with cognition across the AD spectrum.**

| **Metabolites** | **CN** | **EMCI** | **LMCI** | **AD** | **p value** |
| --- | --- | --- | --- | --- | --- |
| M_VLDL_P | 1.26±1.48 | 1.48±1.39 | 1.39±1.24 | 1.22±0.93 | 0.886 |
| S_VLDL_P | 1.25±1.64 | 1.65±1.93 | 1.23±0.88 | 1.30±1.12 | 0.697 |
| XS_VLDL_P | 1.30±1.04 | 1.60±2.39 | 1.11±0.97 | 1.39±1.64 | 0.694 |
| IDL_P | 1.28±0.93 | 1.44±1.73 | 1.16±0.92 | 1.38±1.55 | 0.858 |
| L_LDL_P | 1.25±0.90 | 1.49±1.84 | 1.21±1.02 | 1.29±1.20 | 0.840 |
| M_LDL_P | 1.24±0.88 | 1.57±1.98 | 1.22±1.05 | 1.23±0.96 | 0.668 |
| S_LDL_P | 1.25±0.91 | 1.58±1.98 | 1.19±0.97 | 1.22±0.98 | 0.629 |
| L_HDL_P | 1.10±0.62 | 1.37±0.85 | 1.32±0.77 | 1.22±0.66 | 0.362 |
| M_HDL_P | 1.16±0.68 | 1.37±1.30 | 1.19±0.62 | 1.28±0.62 | 0.735 |
| S_HDL_P | 1.39±1.14 | 1.05±0.76 | 1.14±0.76 | 1.42±1.18 | 0.402 |
| SERUM_C | 1.29±0.96 | 1.38±1.43 | 1.18±0.85 | 1.38±1.70 | 0.924 |
| VLDL_C | 1.28±1.46 | 1.60±2.42 | 1.27±0.92 | 1.30±1.20 | 0.834 |
| REMNANT_C | 1.26±0.96 | 1.59±2.56 | 1.21±0.95 | 1.35±1.54 | 0.781 |
| LDL_C | 1.24±0.87 | 1.55±1.84 | 1.19±0.99 | 1.25±1.02 | 0.657 |
| HDL_C | 1.11±0.78 | 1.32±1.01 | 1.38±0.70 | 1.25±0.67 | 0.488 |
| HDL2_C | 1.13±0.86 | 1.33±1.02 | 1.37±0.68 | 1.25±0.68 | 0.599 |
| HDL3_C | 1.18±0.77 | 1.25±0.83 | 1.34±0.84 | 1.17±0.51 | 0.846 |
| ESTC | 1.27±0.96 | 1.38±1.43 | 1.23±0.92 | 1.36±1.63 | 0.963 |
| FREEC | 1.28±0.92 | 1.36±1.47 | 1.20±0.91 | 1.43±1.85 | 0.924 |
| SERUM_TG | 1.31±1.60 | 1.43±1.34 | 1.36±1.22 | 1.23±1.01 | 0.966 |
| VLDL_TG | 1.27±1.49 | 1.40±1.17 | 1.48±1.39 | 1.21±0.94 | 0.877 |
| LDL_TG | 1.40±1.99 | 1.31±1.44 | 1.25±1.07 | 1.48±1.83 | 0.968 |
| HDL_TG | 1.21±0.63 | 1.24±0.65 | 1.10±0.67 | 1.29±0.78 | 0.794 |
| TOTPG | 1.35±1.11 | 1.23±0.86 | 1.30±1.44 | 1.32±1.67 | 0.986 |
| PC | 1.34±1.07 | 1.23±0.86 | 1.27±1.33 | 1.34±1.72 | 0.982 |
| SM | 1.30±0.99 | 1.30±1.18 | 1.24±0.92 | 1.34±1.69 | 0.992 |
| TOTCHO | 1.38±1.00 | 1.27±0.88 | 1.13±1.14 | 1.36±1.78 | 0.848 |
| TOTFA | 1.30±1.35 | 1.39±1.48 | 1.28±0.96 | 1.32±1.40 | 0.992 |
| DHA | 1.39±1.80 | 1.15±0.68 | 1.39±1.39 | 1.36±1.38 | 0.913 |
| LA | 1.20±0.90 | 1.43±1.41 | 1.25±0.93 | 1.39±1.47 | 0.815 |
| FAW3 | 1.36±1.74 | 1.21±0.74 | 1.36±1.27 | 1.37±1.40 | 0.970 |
| FAW6 | 1.23±0.96 | 1.40±1.36 | 1.21±0.87 | 1.40±1.55 | 0.878 |
| PUFA | 1.27±1.11 | 1.40±1.26 | 1.17±0.78 | 1.40±1.60 | 0.866 |
| MUFA | 1.32±1.56 | 1.54±1.76 | 1.24±0.93 | 1.26±1.18 | 0.878 |
| SFA | 1.37±1.39 | 1.32±1.34 | 1.22±0.85 | 1.30±1.40 | 0.972 |
| VLDL | 11.78±10.09 | 14.62±17.64 | 11.38±8.75 | 12.88±9.91 | 0.735 |
| LDL | 6.12±4.64 | 7.31±8.94 | 5.73±4.77 | 6.47±5.67 | 0.791 |
| HDL | 7.63±3.75 | 8.58±5.36 | 8.33±4.02 | 8.88±3.46 | 0.619 |

**Note:** All data were standardized according to the formula for change in Z value as: Z = (x-μ)/ σ, where x was the data value, μ was the mean value, and σ the standard deviation. For simplicity of calculation, z values were then transformed using an inverse logarithm algorithm with a base of 2. Considering the opposite effects of HDL compared with LDL or VLDL, HDL data were further converted into reciprocal form. P values were obtained from one-way ANOVA; data are presented as means ± standard deviation. **Abbreviations**: VLDL, very low density lipoprotein; IDL, intermediate density lipoprotein; LDL, low density lipoprotein; HDL, high density lipoprotein; M_VLDL_P, total lipids, phospholipids, total cholesterol, cholesterol esters, free cholesterol and triglycerides in medium VLDL particles; S_VLDL_P, total lipids, phospholipids, total cholesterol, cholesterol esters, free cholesterol and triglycerides in small VLDL particles; XS_VLDL_P, total lipids, phospholipids, total cholesterol, cholesterol esters, free cholesterol and triglycerides in very small VLDL particles; IDL_P, total lipids, phospholipids, total cholesterol, cholesterol esters, free cholesterol and triglycerides in IDL particles; L_LDL_P, total lipids, phospholipids, total cholesterol, cholesterol esters, free cholesterol and triglycerides in large LDL particles; M_LDL_P, total lipids, phospholipids, total cholesterol, cholesterol esters, free cholesterol and triglycerides in medium LDL particles; S_LDL_P, total lipids, phospholipids, total cholesterol, cholesterol esters, free cholesterol and triglycerides in small LDL particles; L_HDL_P, total lipids, phospholipids, total cholesterol, cholesterol esters, free cholesterol and triglycerides in large HDL particles; M_HDL_P, total lipids, phospholipids, total cholesterol, cholesterol esters, free cholesterol and triglycerides in medium HDL particles; S_HDL_P, total lipids, phospholipids, total cholesterol, cholesterol esters, free cholesterol and triglycerides in small HDL particles; SERUM_C, serum total cholesterol; VLDL_C, total cholesterol in VLDL; REMNANT_C, remnant cholesterol (non-HDL, non-LDL cholesterol); LDL_C, total cholesterol in LDL; HDL_C, total cholesterol in HDL; HDL2_C, total cholesterol in HDL2; HDL3_C, total cholesterol in HDL3; ESTC, esterified cholesterol; FREEC, free cholesterol; SERUM_TG, serum total triglycerides; VLDL_TG, triglycerides in VLDL; LDL_TG, Triglycerides in LDL; HDL_TG, triglycerides in HDL; TOTPG, total phosphoglycerides; PC, phosphatidylcholine and other cholines; SM, sphingomyelins; TOTCHO, total cholines; TOTFA, total fatty acids; DHA, docosahexaenoic acid; LA, linoleic acid; FAW3, omega-3 fatty acids; FAW6, omega-6 fatty acids; PUFA, polyunsaturated fatty acids; MUFA, monounsaturated fatty acids; SFA, saturated fatty acids.

**Additional file 1: Table S3 Anatomical locations of the 226 regions of interest used to characterize the 10 resting-state networks.**

| **Network** | **ROI** | **MNI coordinations (x, y, z)** | | | **Network** | **ROI** | **MNI coordinations (x, y, z)** | | |
| --- | --- | --- | --- | --- | --- | --- | --- | --- | --- |
| **Auditory** | 61 | 32 | -26 | 13 | **Fronto-parietal** | 195 | -42 | -55 | 45 |
|  | 62 | 65 | -33 | 20 |  | 196 | 40 | 18 | 40 |
|  | 63 | 58 | -16 | 7 |  | 197 | -34 | 55 | 4 |
|  | 64 | -38 | -33 | 17 |  | 198 | -42 | 45 | -2 |
|  | 65 | -60 | -25 | 14 |  | 199 | 33 | -53 | 44 |
|  | 66 | -49 | -26 | 5 |  | 200 | 43 | 49 | -2 |
|  | 67 | 43 | -23 | 20 |  | 201 | -42 | 25 | 30 |
|  | 68 | -50 | -34 | 26 |  | 202 | -3 | 26 | 44 |
|  | 69 | -53 | -22 | 23 | **Salience** | 203 | 11 | -39 | 50 |
|  | 70 | -55 | -9 | 12 |  | 204 | 55 | -45 | 37 |
|  | 71 | 56 | -5 | 13 |  | 205 | 42 | 0 | 47 |
|  | 72 | 59 | -17 | 29 |  | 206 | 31 | 33 | 26 |
|  | 73 | -30 | -27 | 12 |  | 207 | 48 | 22 | 10 |
| **Cingulo-opercular** | 47 | -3 | 2 | 53 |  | 208 | -35 | 20 | 0 |
|  | 48 | 54 | -28 | 34 |  | 209 | 36 | 22 | 3 |
|  | 49 | 19 | -8 | 64 |  | 210 | 37 | 32 | -2 |
|  | 50 | -16 | -5 | 71 |  | 211 | 34 | 16 | -8 |
|  | 51 | -10 | -2 | 42 |  | 212 | -11 | 26 | 25 |
|  | 52 | 37 | 1 | -4 |  | 213 | -1 | 15 | 44 |
|  | 53 | 13 | -1 | 70 |  | 214 | -28 | 52 | 21 |
|  | 54 | 7 | 8 | 51 |  | 215 | 0 | 30 | 27 |
|  | 55 | -45 | 0 | 9 |  | 216 | 5 | 23 | 37 |
|  | 56 | 49 | 8 | -1 |  | 217 | 10 | 22 | 27 |
|  | 57 | -34 | 3 | 4 |  | 218 | 31 | 56 | 14 |
|  | 58 | -51 | 8 | -2 |  | 219 | 26 | 50 | 27 |
|  | 59 | -5 | 18 | 34 |  | 220 | -39 | 51 | 17 |
|  | 60 | 36 | 10 | 1 | **Sensory** | 13 | -7 | -52 | 61 |
| **Dorsal attention** | 251 | 10 | -62 | 61 |  | 14 | -14 | -18 | 40 |
|  | 252 | -52 | -63 | 5 |  | 15 | 0 | -15 | 47 |
|  | 256 | 22 | -65 | 48 |  | 16 | 10 | -2 | 45 |
|  | 257 | 46 | -59 | 4 |  | 17 | -7 | -21 | 65 |
|  | 258 | 25 | -58 | 60 |  | 18 | -7 | -33 | 72 |
|  | 259 | -33 | -46 | 47 |  | 19 | 13 | -33 | 75 |
|  | 260 | -27 | -71 | 37 |  | 20 | -54 | -23 | 43 |
|  | 261 | -32 | -1 | 54 |  | 21 | 29 | -17 | 71 |
|  | 262 | -42 | -60 | -9 |  | 22 | 10 | -46 | 73 |
|  | 263 | -17 | -59 | 64 |  | 23 | -23 | -30 | 72 |
|  | 264 | 29 | -5 | 54 |  | 24 | -40 | -19 | 54 |
| **DMN** | 74 | -41 | -75 | 26 |  | 25 | 29 | -39 | 59 |
|  | 75 | 6 | 67 | -4 |  | 26 | 50 | -20 | 42 |
|  | 76 | 8 | 48 | -15 |  | 27 | -38 | -27 | 69 |
|  | 77 | -13 | -40 | 1 |  | 28 | 20 | -29 | 60 |
|  | 78 | -18 | 63 | -9 |  | 29 | 44 | -8 | 57 |
|  | 79 | -46 | -61 | 21 |  | 30 | -29 | -43 | 61 |
|  | 80 | 43 | -72 | 28 |  | 31 | 10 | -17 | 74 |
|  | 81 | -44 | 12 | -34 |  | 32 | 22 | -42 | 69 |
|  | 82 | 46 | 16 | -30 |  | 33 | -45 | -32 | 47 |
|  | 83 | -68 | -23 | -16 |  | 34 | -21 | -31 | 61 |
|  | 86 | -44 | -65 | 35 |  | 35 | -13 | -17 | 75 |
|  | 87 | -39 | -75 | 44 |  | 36 | 42 | -20 | 55 |
|  | 88 | -7 | -55 | 27 |  | 37 | -38 | -15 | 69 |
|  | 89 | 6 | -59 | 35 |  | 38 | -16 | -46 | 73 |
|  | 90 | -11 | -56 | 16 |  | 39 | 2 | -28 | 60 |
|  | 91 | -3 | -49 | 13 |  | 40 | 3 | -17 | 58 |
|  | 92 | 8 | -48 | 31 |  | 41 | 38 | -17 | 45 |
|  | 93 | 15 | -63 | 26 |  | 42 | -49 | -11 | 35 |
|  | 94 | -2 | -37 | 44 |  | 43 | 36 | -9 | 14 |
|  | 95 | 11 | -54 | 17 |  | 44 | 51 | -6 | 32 |
|  | 96 | 52 | -59 | 36 |  | 45 | -53 | -10 | 24 |
|  | 97 | 23 | 33 | 48 |  | 46 | 66 | -8 | 25 |
|  | 98 | -10 | 39 | 52 |  | 255 | 47 | -30 | 49 |
|  | 99 | -16 | 29 | 53 | **Subcortical** | 222 | 6 | -24 | 0 |
|  | 100 | -35 | 20 | 51 |  | 223 | -2 | -13 | 12 |
|  | 101 | 22 | 39 | 39 |  | 224 | -10 | -18 | 7 |
|  | 102 | 13 | 55 | 38 |  | 225 | 12 | -17 | 8 |
|  | 103 | -10 | 55 | 39 |  | 226 | -5 | -28 | -4 |
|  | 104 | -20 | 45 | 39 |  | 227 | -22 | 7 | -5 |
|  | 105 | 6 | 54 | 16 |  | 228 | -15 | 4 | 8 |
|  | 106 | 6 | 64 | 22 |  | 229 | 31 | -14 | 2 |
|  | 107 | -7 | 51 | -1 |  | 230 | 23 | 10 | 1 |
|  | 108 | 9 | 54 | 3 |  | 231 | 29 | 1 | 4 |
|  | 109 | -3 | 44 | -9 |  | 232 | -31 | -11 | 0 |
|  | 110 | 8 | 42 | -5 |  | 233 | 15 | 5 | 7 |
|  | 111 | -11 | 45 | 8 |  | 234 | 9 | -4 | 6 |
|  | 112 | -2 | 38 | 36 | **Ventral attention** | 235 | 54 | -43 | 22 |
|  | 113 | -3 | 42 | 16 |  | 236 | -56 | -50 | 10 |
|  | 114 | -20 | 64 | 19 |  | 237 | -55 | -40 | 14 |
|  | 115 | -8 | 48 | 23 |  | 238 | 52 | -33 | 8 |
|  | 116 | 65 | -12 | -19 |  | 239 | 51 | -29 | -4 |
|  | 117 | -56 | -13 | -10 |  | 240 | 56 | -46 | 11 |
|  | 118 | -58 | -30 | -4 |  | 241 | 53 | 33 | 1 |
|  | 119 | 65 | -31 | -9 |  | 242 | -49 | 25 | -1 |
|  | 120 | -68 | -41 | -5 | **Visual** | 143 | 18 | -47 | -10 |
|  | 121 | 13 | 30 | 59 |  | 144 | 40 | -72 | 14 |
|  | 122 | 12 | 36 | 20 |  | 145 | 8 | -72 | 11 |
|  | 123 | 52 | -2 | -16 |  | 146 | -8 | -81 | 7 |
|  | 124 | -26 | -40 | -8 |  | 147 | -28 | -79 | 19 |
|  | 125 | 27 | -37 | -13 |  | 148 | 20 | -66 | 2 |
|  | 126 | -34 | -38 | -16 |  | 149 | -24 | -91 | 19 |
|  | 127 | 28 | -77 | -32 |  | 150 | 27 | -59 | -9 |
|  | 128 | 52 | 7 | -30 |  | 151 | -15 | -72 | -8 |
|  | 129 | -53 | 3 | -27 |  | 152 | -18 | -68 | 5 |
|  | 130 | 47 | -50 | 29 |  | 153 | 43 | -78 | -12 |
|  | 131 | -49 | -42 | 1 |  | 154 | -47 | -76 | -10 |
|  | 137 | -46 | 31 | -13 |  | 155 | -14 | -91 | 31 |
|  | 139 | 49 | 35 | -12 |  | 156 | 15 | -87 | 37 |
| **Fronto-parietal** | 174 | -44 | 2 | 46 |  | 157 | 29 | -77 | 25 |
|  | 175 | 48 | 25 | 27 |  | 158 | 20 | -86 | -2 |
|  | 176 | -47 | 11 | 23 |  | 159 | 15 | -77 | 31 |
|  | 177 | -53 | -49 | 43 |  | 160 | -16 | -52 | -1 |
|  | 178 | -23 | 11 | 64 |  | 161 | 42 | -66 | -8 |
|  | 179 | 58 | -53 | -14 |  | 162 | 24 | -87 | 24 |
|  | 180 | 24 | 45 | -15 |  | 163 | 6 | -72 | 24 |
|  | 181 | 34 | 54 | -13 |  | 164 | -42 | -74 | 0 |
|  | 186 | 47 | 10 | 33 |  | 165 | 26 | -79 | -16 |
|  | 187 | -41 | 6 | 33 |  | 166 | -16 | -77 | 34 |
|  | 188 | -42 | 38 | 21 |  | 167 | -3 | -81 | 21 |
|  | 189 | 38 | 43 | 15 |  | 168 | -40 | -88 | -6 |
|  | 190 | 49 | -42 | 45 |  | 169 | 37 | -84 | 13 |
|  | 191 | -28 | -58 | 48 |  | 170 | 6 | -81 | 6 |
|  | 192 | 44 | -53 | 47 |  | 171 | -26 | -90 | 3 |
|  | 193 | 32 | 14 | 56 |  | 172 | -33 | -79 | -13 |
|  | 194 | 37 | -65 | 40 |  | 173 | 37 | -81 | 1 |

MNI = Montreal Neurological Institute; ROI = region of interest.

**Additional file 1: Table S4 Canonical correlation coefficients and p values within- and between the ten predefined RSNs.**

| Network variables | r | log_10_(p) values | Network variables | r | log_10_(p) value |
| --- | --- | --- | --- | --- | --- |
| AUD | 0.98785 | -37.39903 | DAN-DMN | 0.99663 | -57.4023 |
| CON | 0.99169 | -43.19723 | DAN-FPN | 0.99408 | -48.48413 |
| DAN | 0.99051 | -41.16431 | DAN-SAN | 0.99422 | -48.84466 |
| DMN | 0.99216 | -44.11295 | DAN-SMN | 0.99546 | -52.65956 |
| FPN | 0.98356 | -32.86328 | DAN-SUB | 0.98943 | -39.51713 |
| SAN | 0.9928 | -45.42713 | DAN-VAN | 0.99513 | -51.54516 |
| SMN | 0.99015 | -40.59346 | DAN-AUD | 0.98822 | -37.86328 |
| SUB | 0.99589 | -54.23359 | DMN-FPN | 0.9961 | -55.05257 |
| VAN | 0.99029 | -40.81248 | DMN-SAN | 0.99572 | -53.60555 |
| AUD | 0.96616 | -22.46344 | DMN-SMN | 0.99447 | -49.54212 |
| AUD-CON | 0.99512 | -51.5157 | DMN-SUB | 0.99442 | -49.41341 |
| AUD-DAN | 0.99492 | -50.88273 | DMN-VAN | 0.99448 | -49.58336 |
| AUD-DMN | 0.99661 | -57.29243 | DMN-AUD | 0.99425 | -48.9431 |
| AUD-FPN | 0.99578 | -53.81816 | FPN-SAN | 0.99573 | -53.64397 |
| AUD-SAN | 0.99435 | -49.21968 | FPN-SMN | 0.99607 | -54.95861 |
| AUD-SMN | 0.99433 | -49.16749 | FPN-SUB | 0.98757 | -37.04624 |
| AUD-SUB | 0.99031 | -40.84466 | FPN-VAN | 0.99395 | -48.12726 |
| AUD-VAN | 0.99147 | -42.7986 | FPN-AUD | 0.98602 | -35.28316 |
| AUD-AUD | 0.98758 | -37.06248 | SAN-SMN | 0.99613 | -55.20691 |
| CON- DAN | 0.99497 | -51.02872 | SAN-SUB | 0.9937 | -47.51145 |
| CON-DMN | 0.99738 | -61.42136 | SAN-VAN | 0.99395 | -48.1463 |
| CON-FPN | 0.99442 | -49.41908 | SAN-AUD | 0.98864 | -38.42022 |
| CON-SAN | 0.99652 | -56.86328 | SMN-SUB | 0.99023 | -40.70997 |
| CON-SMN | 0.99699 | -59.17849 | SMN-VAN | 0.99237 | -44.52143 |
| CON-SUB | 0.99209 | -43.97469 | SMN-AUD | 0.98866 | -38.4389 |
| CON-VAN | 0.9943 | -49.0752 | SUB-VAN | 0.98977 | -40.00833 |
| CON-AUD | 0.9909 | -41.80688 | SUB-AUD | 0.98912 | -39.07366 |
|  |  |  | VAN-AUD | 0.98777 | -37.29414 |

Notes: RSNs, resting-state networks; AUD, the auditory network; CON, the cingulo-opercular network; DAN, the dorsal attention network; DMN the default mode network; FPN, the fronto-parietal network; SAN, the salience network; SMN the sensory network; SUB, the subcortical network; VAN, the ventral attention network; VIS, the visual network.

**Additional file 1: Table S5 Canonical correlation coefficients and p values of lipid-related genetic scores and lipoproteins.**

| Lipid variables | r | log10(p) value | Lipid variables | r | log10(p) value |
| --- | --- | --- | --- | --- | --- |
| VLDL | 1.00000 | -299.00000 | **HDL3_C** | 1.00000 | -299.00000 |
| LDL | 1.00000 | -299.00000 | **ESTC** | 1.00000 | -299.00000 |
| HDL | 1.00000 | -299.00000 | **FREEC** | 1.00000 | -299.00000 |
| sumM_VLDL_P | 1.00000 | -299.00000 | **SERUM_TG** | 0.99989 | -129.75696 |
| sumS_VLDL_P | 1.00000 | -299.00000 | **VLDL_TG** | 1.00000 | -299.00000 |
| sumXS_VLDL_P | 1.00000 | -299.00000 | **LDL_TG** | 1.00000 | -299.00000 |
| sumIDL_P | 0.99990 | -131.27327 | **HDL_TG** | 1.00000 | -299.00000 |
| sumL_LDL_P | 1.00000 | -299.00000 | **TOTPG** | 0.99924 | -94.54061 |
| sumM_LDL_P | 1.00000 | -299.00000 | **PC** | 1.00000 | -265.42829 |
| sumS_LDL_P | 1.00000 | -299.00000 | **SM** | 1.00000 | -269.77728 |
| sumL_HDL_P | 1.00000 | -299.00000 | **TOTCHO** | 1.00000 | -263.41229 |
| sumM_HDL_P | 1.00000 | -299.00000 | **TOTFA** | 0.99995 | -145.30452 |
| sumS_HDL_P | 1.00000 | -299.00000 | **DHA** | 0.99759 | -73.92812 |
| SERUM_C | 1.00000 | -299.00000 | **LA** | 0.99883 | -86.89963 |
| VLDL_C | 1.00000 | -299.00000 | **FAW3** | 0.99872 | -85.26761 |
| REMNANT_C | 0.99953 | -103.24109 | **FAW6** | 0.99993 | -137.05799 |
| LDL_C | 1.00000 | -299.00000 | **PUFA** | 0.99992 | -134.65170 |
| HDL_C | 1.00000 | -299.00000 | **MUFA** | 0.99951 | -102.53165 |
| HDL2_C | 1.00000 | -299.00000 | **SFA** | 0.99970 | -111.50446 |

Notes: Abbreviation of lipoproteins were described in the **Supplementary Table** 2.

**Additional file 1: Table S6 Post hoc analysis revealed correlation coefficients and p values among lipid-related genetic scores and lipoproteins, large-scale network connectivity, CSF biomarkers, and cognitive performance**

| Network variables | LDL | | sumIDL_P | | sumL_LDL_P | | LDL_C | | HDL_TG | | TOTPG | |
| --- | --- | --- | --- | --- | --- | --- | --- | --- | --- | --- | --- | --- |
|  | **r** | **p** | **r** | **p** | **r** | **p** | **r** | **p** | **r** | **p** | **r** | **p** |
| CON | 0.304 | 0.064 | 0.330 | 0.043 | 0.335 | 0.040 | 0.320 | 0.050 |  |  |  |  |
| VAN | 0.144 | 0.388 |  |  |  |  |  |  |  |  | 0.323 | 0.048 |
| AUD-DAN | 0.233 | 0.158 |  |  |  |  |  |  | -0.321 | 0.049 |  |  |
| AUD-VAN |  | 0.824 |  |  |  |  |  |  |  |  |  |  |
| AUD-VIS | 0.090 | 0.591 |  |  |  |  |  |  | -0.340 | 0.037 |  |  |
| CON-DAN | 0.333 | 0.041 | 0.364 | 0.025 | 0.356 | 0.028 | 0.340 | 0.037 |  |  |  |  |
| CON-DMN | 0.402 | 0.012 | 0.418 | 0.009 | 0.425 | 0.008 | 0.414 | 0.010 |  |  | 0.326 | 0.046 |
| CON-SAN |  |  | 0.335 | 0.040 |  |  |  |  |  |  |  |  |
| CON-SUB | 0.320 | 0.050 | 0.338 | 0.038 | 0.359 | 0.027 | 0.361 | 0.026 |  |  |  |  |
| CON-VAN |  |  |  |  |  |  |  |  | -0.403 | 0.012 |  |  |
| CON-VIS |  |  |  |  |  |  |  |  | -0.336 | 0.039 |  |  |
| DAN-DMN | 0.353 | 0.030 | 0.348 | 0.032 | 0.358 | 0.027 | 0.352 | 0.030 |  |  | 0.333 | 0.041 |
| DAN-SAN |  |  | 0.367 | 0.024 | 0.335 | 0.039 |  |  |  |  | 0.372 | 0.021 |
| DAN-SUB | 0.334 | 0.041 | 0.355 | 0.029 | 0.364 | 0.024 | 0.360 | 0.027 |  |  |  |  |
| DAN-VAN |  |  |  |  |  |  |  |  |  |  |  |  |
| DMN-SAN |  |  | 0.321 | 0.049 |  |  |  |  |  |  | 0.377 | 0.020 |
| DMN-SUB | 0.395 | 0.014 | 0.400 | 0.013 | 0.420 | 0.009 | 0.417 | 0.009 |  |  |  |  |
| DMN-VAN |  |  |  |  |  |  |  |  |  |  | 0.345 | 0.034 |
| FPN-SUB |  |  | 0.323 | 0.048 |  |  |  |  |  |  |  |  |
| FPN-VAN |  |  |  |  |  |  |  |  | -0.324 | 0.047 |  |  |
| SAN-VAN |  |  |  |  |  |  |  |  | -0.334 | 0.041 | 0.354 | 0.029 |
| SMN-VAN |  |  |  |  |  |  |  |  | -0.341 | 0.036 |  |  |
| SMN-VIS |  |  |  |  |  |  |  |  | -0.335 | 0.040 |  |  |
| Network variables | **sumM_HDL_P** | | **PC** | | **TOTCHO** | | **LA** | | **HDL** | | **sumM_LDL_P** | |
|  | **r** | **p** | **r** | **p** | **r** | **p** | **r** | **p** | **r** | **p** | **r** | **p** |
| AUD-VAN | -0.341 | 0.036 |  |  |  |  |  |  |  |  |  |  |
| CON-DAN |  |  |  |  |  |  |  |  |  |  | 0.323 | 0.048 |
| CON-DMN |  |  | 0.328 | 0.045 | 0.329 | 0.044 | 0.346 | 0.033 |  |  | 0.398 | 0.013 |
| CON-SUB |  |  |  |  |  |  |  |  |  |  | 0.341 | 0.036 |
| CON-VAN |  |  |  |  |  |  |  |  | -0.356 | 0.028 |  |  |
| DAN-DMN |  |  | 0.333 | 0.041 | 0.324 | 0.047 |  |  |  |  | 0.346 | 0.034 |
| DAN-SAN |  |  | 0.374 | 0.021 | 0.370 | 0.022 | 0.325 | 0.046 |  |  |  |  |
| DAN-SUB |  |  |  |  |  |  |  |  |  |  | 0.344 | 0.035 |
| DAN-VAN | -0.335 | 0.040 |  |  |  |  |  |  |  |  |  |  |
| DMN-SAN |  |  | 0.375 | 0.020 | 0.370 | 0.022 | 0.332 | 0.042 |  |  |  |  |
| DMN-SUB |  |  | 0.321 | 0.049 |  |  |  |  |  |  | 0.405 | 0.012 |
| DMN-VAN |  |  | 0.339 | 0.037 | 0.331 | 0.043 |  |  |  |  |  |  |
| SAN-VAN | -0.392 | 0.015 | 0.346 | 0.033 | 0.334 | 0.040 | 0.331 | 0.043 | -0.405 | 0.012 |  |  |
| SUB-VAN | -0.400 | 0.013 |  |  |  |  |  |  | -0.456 | 0.004 |  |  |
| Network variables | **sumS_LDL_P** | | **SERUM_C** | | **VLDL_C** | | **ESTC** | | **FREEC** | | **SM** | |
|  | **r** | **p** | **r** | **p** | **r** | **p** | **r** | **p** | **r** | **p** | **r** | **p** |
| CON-DAN |  |  | **0.352** | **0.030** | **0.322** | **0.048** | **0.354** | **0.029** | **0.346** | **0.033** |  |  |
| CON-DMN | **0.391** | 0.015 | 0.399 | 0.013 | 0.333 | 0.041 | 0.402 | 0.012 | 0.392 | 0.015 | 0.373 | 0.021 |
| CON-SAN |  |  | 0.330 | 0.043 |  |  | 0.331 | 0.042 | 0.327 | 0.045 | 0.324 | 0.047 |
| CON-SUB | **0.321** | 0.049 |  |  |  |  |  |  |  |  |  |  |
| DAN-DMN | **0.346** | 0.034 | 0.354 | 0.029 |  |  | 0.358 | 0.027 | 0.343 | 0.035 |  |  |
| DAN-SAN |  |  | 0.382 | 0.018 |  |  | 0.385 | 0.017 | 0.374 | 0.021 | 0.370 | 0.022 |
| DAN-SUB | **0.339** | 0.037 | 0.336 | 0.039 |  |  | 0.341 | 0.036 | 0.324 | 0.047 |  |  |
| DMN-SAN |  |  | 0.354 | 0.029 |  |  | 0.357 | 0.028 | 0.345 | 0.034 | 0.356 | 0.028 |
| DMN-SUB | **0.397** | 0.014 | 0.382 | 0.018 | 0.328 | 0.045 | 0.387 | 0.016 | 0.370 | 0.022 | 0.342 | 0.036 |

Notes: Abbreviation of lipoproteins and networks were described in the Supplementary Table 2 and 4.

**Additional file 1: Table S7 Twenty-two functional connection links used for classification between groups.**

| **Twenty-two functional connections used for classification** | | |
| --- | --- | --- |
| ROI51-ROI65 | ROI188-ROI53 | ROI120-ROI260 |
| ROI252-ROI73 | ROI14-ROI252 | ROI137-ROI95 |
| ROI252-ROI51 | ROI14-ROI263 | ROI137-ROI105 |
| ROI252-ROI53 | ROI16-ROI263 | ROI207-ROI72 |
| ROI124-ROI251 | ROI28-ROI62 | ROI237-ROI191 |
| ROI181-ROI117 | ROI233-ROI42 | ROI164-ROI191 |
| ROI187-ROI47 | ROI239-ROI104 |  |
| ROI187-ROI51 | ROI145-ROI67 |  |

Notes: Nodes of the link were from Power-atlas. **Abbreviations:** ROI = region of interest.

**Additional file 1: Table S8** Modified model fit indices.

| Fit indices | Allowable amount | The obtained value |
| --- | --- | --- |
|  |  |  |
| CMIN/DF | lower than 3 | 1.070 |
| RMSEA | Lower than 0.08 | 0.040 |
| GFI | Greater than 0.8 | 0.817 |
| AGFI | Greater than 0.8 | 0.729 |
| TLI | Greater than 0.9 | 0.988 |
| NFI | Greater than 0.9 | 0.878 |
| CFI | Greater than 0.9 | 0.991 |
| IFI | Greater than 0.9 | 0.991 |

**Abbreviations:** CMIN/DF, chi square/degree of freedom ratio; RMSEA, root-mean-square error of approximation; GFI, goodness-of-fit index; AGFI, adjusted GFI, TLI, Tucker-Lewis Index; NFI, normed fit index; CFI, comparative fit index; IFI, incremental fit index.

**Additional file 1: Table S9** Results of Structural Path model of direct effects.

| Hypothesized paths | | | Unstandardized  path coefficient | S.E. | t-value | p-value |
| --- | --- | --- | --- | --- | --- | --- |
| Dyslipidemia | -- > | Pathology | 0.18 | 0.13 | 1.38 | 0.17 |
| Dyslipidemia | -- > | Brain function 1 | 0.19 | 0.09 | 1.97 | **0.02** |
| Dyslipidemia | -- > | Cognition | 0.33 | 0.24 | 1.37 | 0.14 |
| Pathology | -- > | Brain function 1 | 0.1 | 0.09 | 1.07 | 0.28 |
| Pathology | -- > | Brain function 2 | 0.01 | 0.03 | 0.32 | 0.37 |
| Pathology | -- > | Cognition | 0.81 | 0.27 | 3.02 | **0.00** |
| Brain function 1 | -- > | Brain function 2 | 0.51 | 0.09 | 5.57 | **0.00** |
| Brain function 2 | -- > | Cognition | 0.25 | 0.82 | 0.3 | 0.93 |

**Additional file 1: Fig. S1 Nonlinear curves fitted between cerebrospinal fluid biomarkers and global cognitive performance. Abbreviations:** MMSE, mini-mental state examination; ADAS-cog, Alzheimer’s disease assessment scale-cognitive section; Aβ, amyloid 1 to 42 peptide; Tau, total tau; pTau, tau phosphorylated at the threonine 181 position; CSF, cerebrospinal fluid.


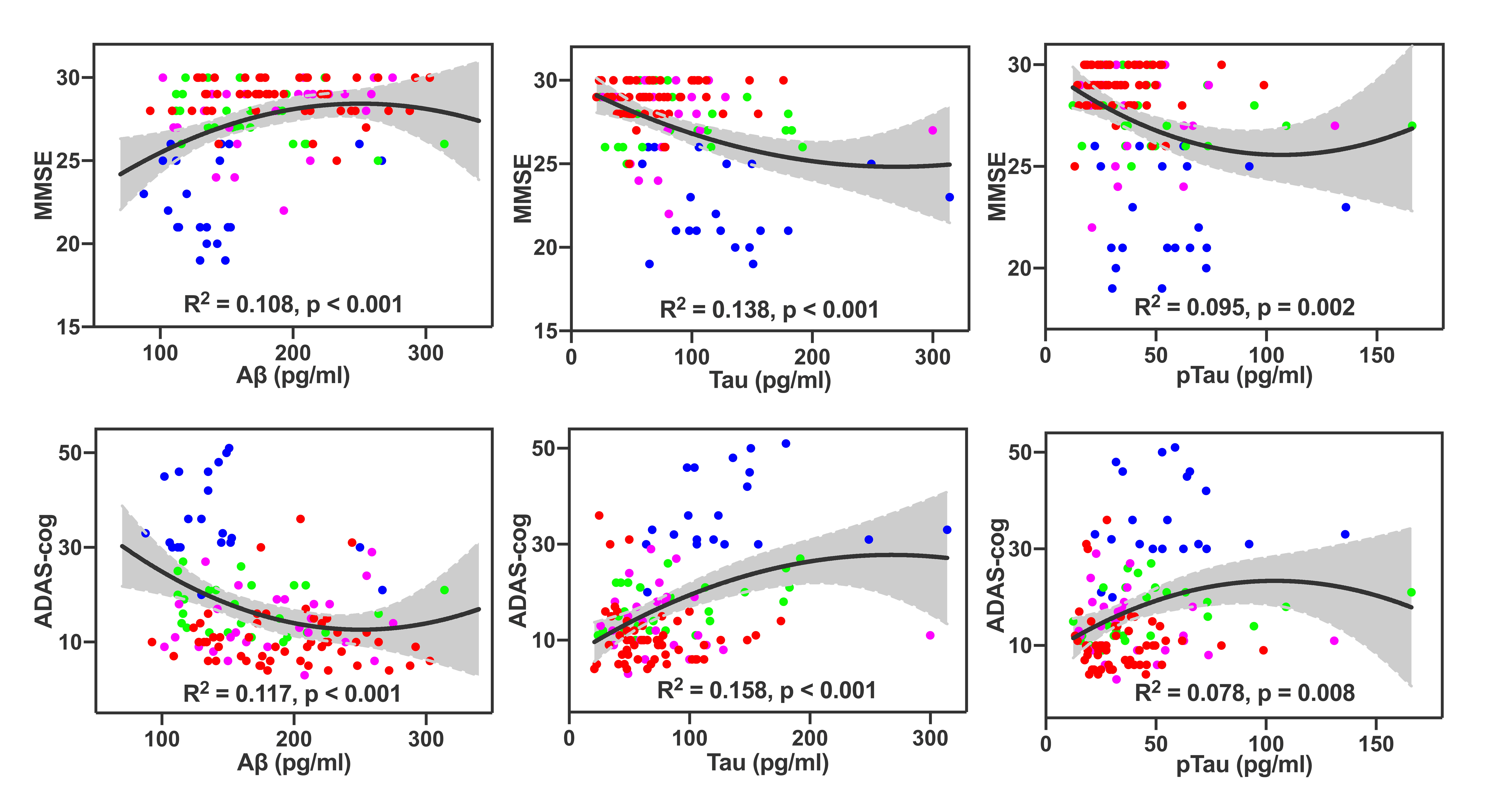


**
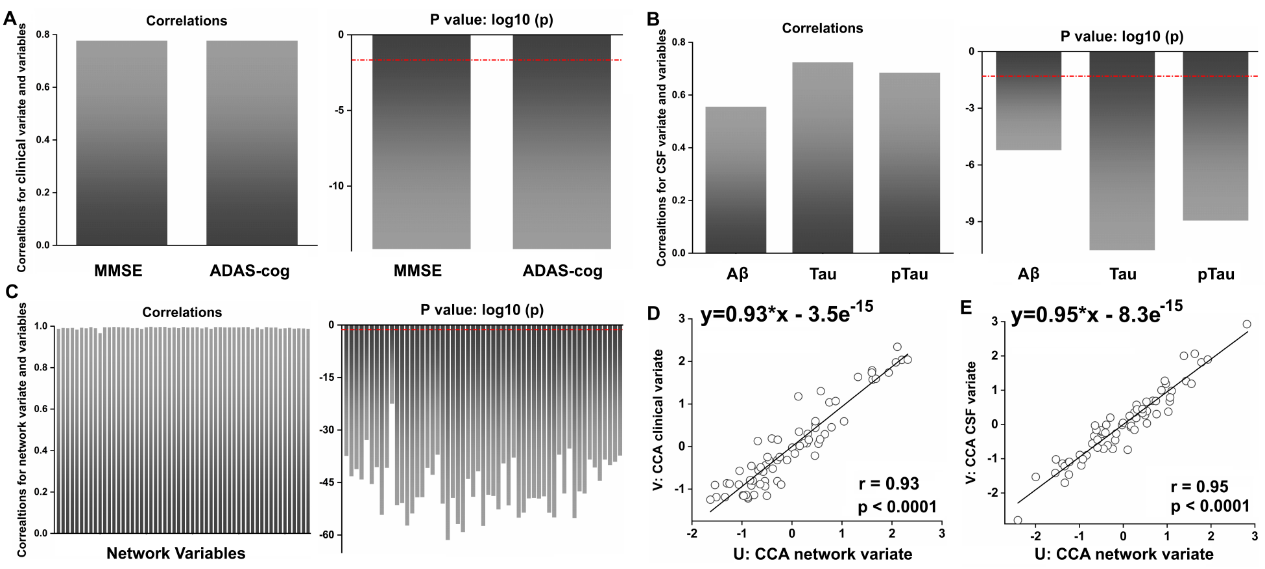
**

**Additional file 1: Fig. S2 Correlations and their significance between the following in patients with EMCI, LMCI and AD:** the two clinical cognitive performance variables and clinical CCA mode (A); the three cerebrospinal fluid biomarker variables and CSF CCA mode (B); fifty-five within and pairwise between-network variables and network CCA mode (C); first pairwise CCA mode (D), and second pairwise CCA mode (E). Note P values in A, B and C, have been log10-transformed. Red dashed lines represent a log10-transformed P value of 0.05. **Abbreviations:** MMSE, mini-mental state examination; ADAS-cog, Alzheimer’s disease assessment scale-cognitive section; Aβ, amyloid 1 to 42 peptide; Tau, total tau; pTau, tau phosphorylated at the threonine 181 position; CSF, cerebrospinal fluid; CCA, canonical correlation analysis.


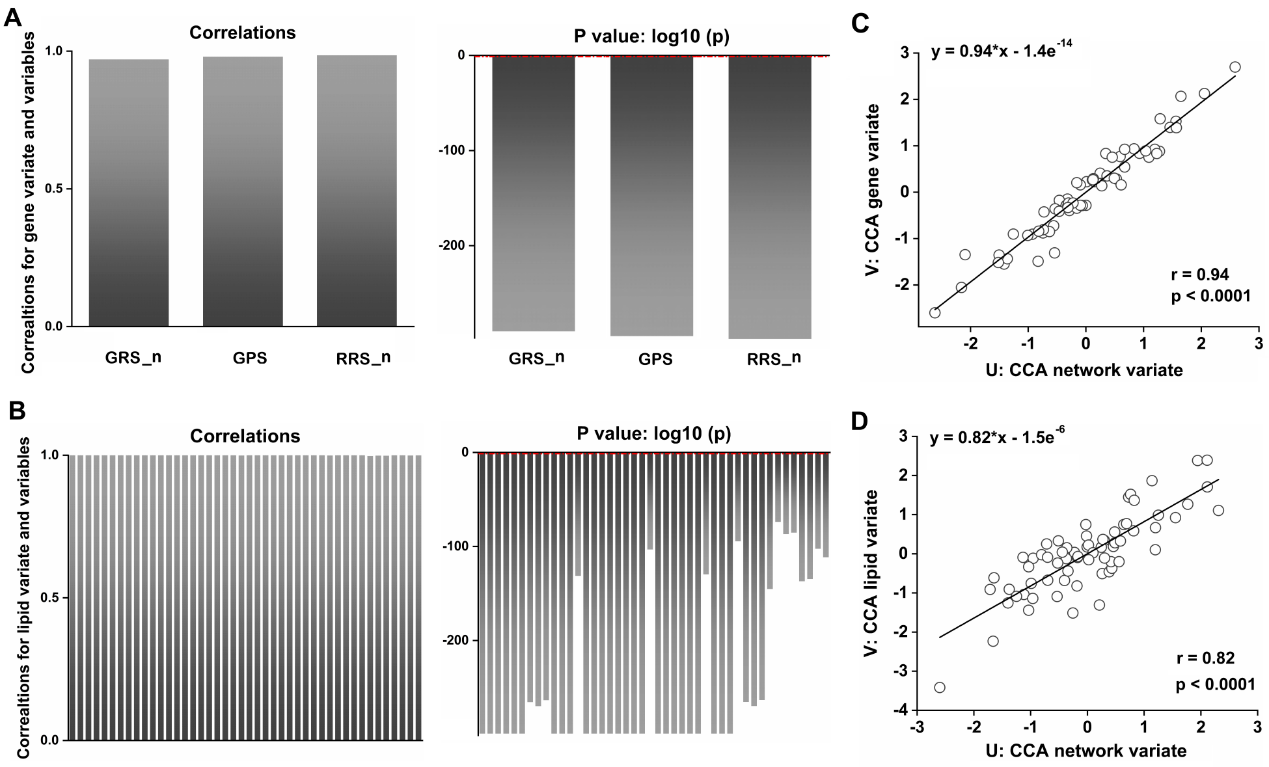
 **Additional file 1: Fig. S3 Correlations and their significance between the following in patients with EMCI, LMCI and AD: t**he three gene score variables and the second gene CCA mode (A), the thirty-eight serum lipid variables and serum lipid CCA mode (B), the fifth pairs of CCA modes (C) and sixth pairs of CCA modes (D). All data for cholesterol metabolites in the blood were z-transformed. **Note** that the P values in A and B were log10-transformed. Red dashed lines represent a log10-transformed P value of 0.05. **Abbreviations:** GPS, genetic protective score; GRS_n, genetic risk score without APOE ε4; RRS, relative risk score without APOE ε4; CCA, canonical correlation analysis.


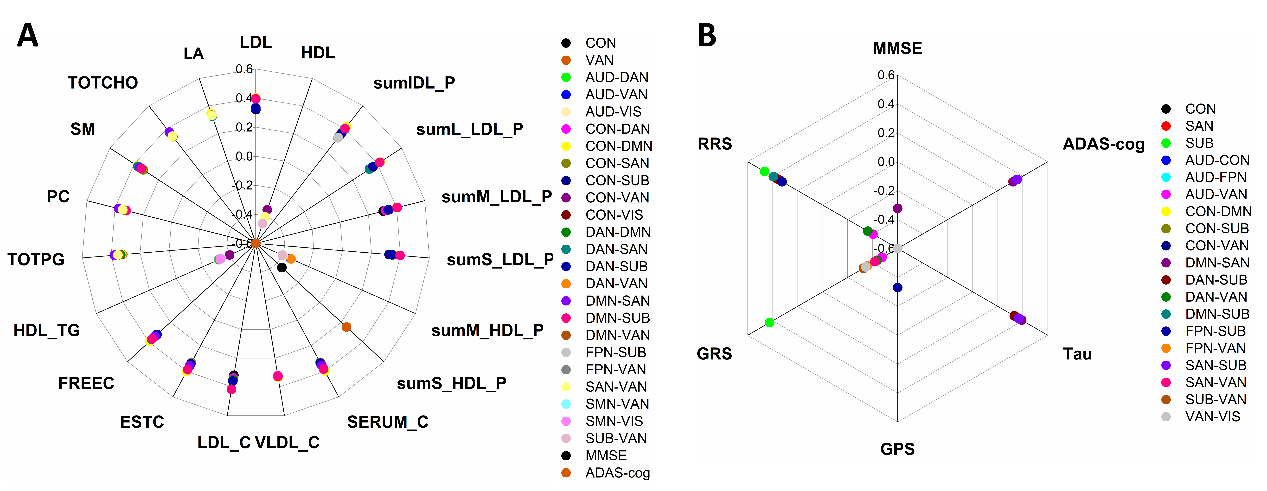


**Additional file 1: Fig. S4 Radar plots indicating patterns of association for serum lipid, cognitive performance, CSF biomarkers, and polygenic scores to network connectivity.** Values displayed by the dots in the radar plots are values of Pearson’s correlation coefficients. All nodes represent a statistically significant correlation coefficient (p value<0.05). Abbreviations: AUD, the auditory network; CON, the cingulo-opercular network; DAN, the dorsal attention network; DMN, the default mode network; FPN, the fronto-parietal network; SAN, the salience network; SMN, the sensory network; SUB, the subcortical network; VAN, the ventral attention network; VIS, the visual network; LDL, low density lipoprotein; MMSE, mini-mental state examination; ADAS-cog, Alzheimer’s disease assessment scale-cognitive section; Tau, total tau; GPS, genetic protective score; GRS, genetic risk score; RRS, relative risk score. The abbreviations of plasma cholesterol metabolites were are provided in Supplementary Table2.

**
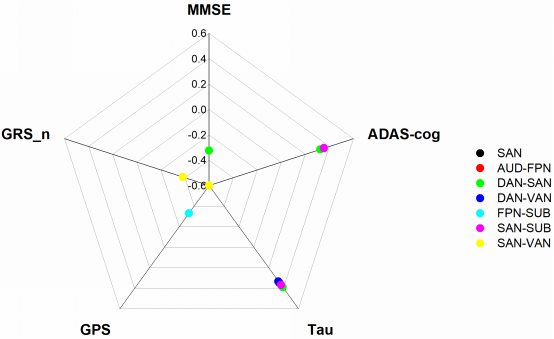
 Additional file 1: Fig. S5 Radar plots demonstrating patterns of association of cognitive performance, CSF biomarkers, and gene scores to network connectivity.** Values displayed by the dots in the radar plots are values of Pearson’s correlation coefficients. All nodes represent a statistically significant correlation coefficient (p value<0.05). **Abbreviations:** AUD, the auditory network; DAN, the dorsal attention network; FPN, the fronto-parietal network; SAN, the salience network; SUB, the subcortical network; VAN, the ventral attention network; MMSE, mini-mental state examination; ADAS-cog, Alzheimer’s disease assessment scale-cognitive section; Tau, total tau; GPS, genetic protective score; GRS_n, genetic risk score without APOE ε4.
